# Cardiovascular and pharmacological implications of haem-deficient NO-unresponsive soluble guanylate cyclase knock-in mice

**DOI:** 10.1038/ncomms9482

**Published:** 2015-10-07

**Authors:** Robrecht Thoonen, Anje Cauwels, Kelly Decaluwe, Sandra Geschka, Robert E. Tainsh, Joris Delanghe, Tino Hochepied, Lode De Cauwer, Elke Rogge, Sofie Voet, Patrick Sips, Richard H. Karas, Kenneth D. Bloch, Marnik Vuylsteke, Johannes-Peter Stasch, Johan Van de Voorde, Emmanuel S. Buys, Peter Brouckaert

**Affiliations:** 1 Laboratory for Molecular Pathology and Experimental Therapy, Inflammation Research Center, VIB, B-9052 Ghent, Belgium; 2 Department of Biomedical Molecular Biology, Ghent University, B-9052 Ghent, Belgium; 3 Department of Pharmacology, Ghent University, B-9000 Ghent, Belgium; 4 Cardiovascular Research, Bayer Pharma AG, D-42096 Wuppertal, Germany; 5 Department of Anesthesia, Critical Care and Pain Medicine, Massachusetts General Hospital Research Institute, Boston, Massachusetts 02114, USA; 6 Department of Clinical Biology, Ghent University Hospital, B-9000 Ghent, Belgium; 7 Molecular Cardiology Research Center, Molecular Cardiology Research Institute, Tufts Medical Center, Boston Massachusetts 02111, USA; 8 Department of Plant Systems Biology, VIB, B-9052 Ghent, Belgium; 9 Department of Plant Biotechnology and Genetics, Ghent University, B-9052 Ghent, Belgium; 10 Department of Pharmacology, The School of Pharmacy, Martin-Luther-University, Halle, Germany

## Abstract

Oxidative stress, a central mediator of cardiovascular disease, results in loss of the prosthetic haem group of soluble guanylate cyclase (sGC), preventing its activation by nitric oxide (NO). Here we introduce Apo-sGC mice expressing haem-free sGC. Apo-sGC mice are viable and develop hypertension. The haemodynamic effects of NO are abolished, but those of the sGC activator cinaciguat are enhanced in apo-sGC mice, suggesting that the effects of NO on smooth muscle relaxation, blood pressure regulation and inhibition of platelet aggregation require sGC activation by NO. Tumour necrosis factor (TNF)-induced hypotension and mortality are preserved in apo-sGC mice, indicating that pathways other than sGC signalling mediate the cardiovascular collapse in shock. Apo-sGC mice allow for differentiation between sGC-dependent and -independent NO effects and between haem-dependent and -independent sGC effects. Apo-sGC mice represent a unique experimental platform to study the *in vivo* consequences of sGC oxidation and the therapeutic potential of sGC activators.

## Abstract (web summary)

Haem-free, NO-insensitive soluble guanylate cyclase (apo-sGC) generated during oxidative stress contributes to cardiovascular pathology. By generating and characterizing apo-sGC knock-in mice, Thoonen *et al*. provide a scientific ground for the therapeutic concept of sGC activators, and dissect the relevance of the NO-sGC axis.

## Graphical abstract

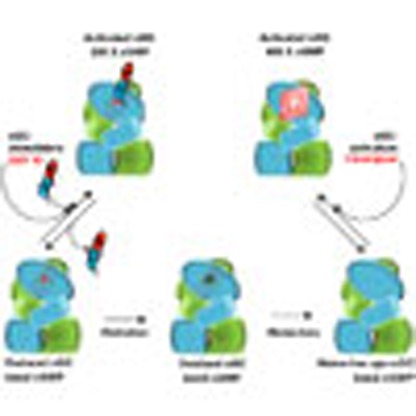

Oxidative stress is a risk factor for cardiovascular disorders^1^,^2^. Oxidative stress interferes with the nitric oxide (NO)/soluble guanylate cyclase (sGC)/3′,5′-cyclic guanosine monophosphate (cGMP) pathway^1^,^2^,^3^, critical for cardiovascular and platelet function. First, reactive oxygen species can uncouple nitric oxide synthase (NOS), resulting in the production of superoxide (O_2_^-·^) instead of NO. Second, O_2_^-·^ scavenges NO, decreasing its bioavailability. Third, oxidative stress impairs the sensitivity of sGC for NO^4^. sGC is a heterodimeric haemoprotein, consisting of an α1 or α2 subunit combined with a common β1 subunit, which generates cGMP. Basal catalytic activity of sGC is greatly enhanced upon binding of NO to the ferrous haem, disrupting the bond between the haem and the HIS^105^ residue of sGCβ1 (ref. 5). Although sGC is considered as the principal target of NO, this paradigm has been challenged by the discovery of signalling pathways involving protein nitr(osyl)ation^6^. Oxidation of the ferrous haem, associated with haem-dissociation *in vitro*, results in the generation of haem-free sGC (apo-sGC), which has preserved basal activity but can no longer be activated by NO^7^,^8^. *In vitro*, apo-sGC has been shown to be susceptible to ubiquitination and subsequent proteasomal breakdown^9^. Levels of apo-sGC are hypothesized to be increased in conditions of oxidative stress *in vivo*^4^,^10^,^11^,^12^.

The development of drugs targeting apo-sGC represents a unique approach to treat pathologies characterized by oxidative stress and associated with impaired cGMP signalling. Two classes of drugs modulating sGC activity exist: NO-independent, haem-dependent sGC stimulators, such as BAY 41-2272 and riociguat (Adempas or BAY 63-2521)^13^, and NO- and haem-independent sGC activators, such as cinaciguat (BAY 58-2667) and ataciguat (HMR1766)^14^,^15^. The therapeutic potential of riociguat was recently illustrated in phase III clinical trials for the treatment of pulmonary arterial hypertension (PAH)^13^ and subsequent Food and Drug Administration approval for PAH treatment. However, the inability to distinguish native, haem-containing sGC from oxidized/haem-free sGC^8^ *in vivo* has precluded investigations into the specific pathological consequences of impaired sGC activity through haem-oxidation *in vivo*, as well as the consequences of specifically activating the oxidized/haem-free pool of sGC *in vivo*. To provide a scientific basis for the concept of targeting apo-sGC as a pharmacological strategy, we generated mice expressing haem-free sGC (apo-sGC mice) instead of native sGC, by mutation of the HIS105 residue of sGCβ1 to PHE105.

Here, we report the *in vivo* cardiovascular phenotype of apo-sGC mice, identifying activation of haem-containing reduced sGC as the essential mechanism by which NO induces vasorelaxation, lowers blood pressure (BP) and inhibits platelet aggregation. Our data suggest that sGC activators, a new class of drugs in development for the treatment of a variety of cardiovascular diseases, can attenuate haemodynamic abnormalities associated with oxidative stress. In addition, apo-sGC mice allow to distinguish between sGC-dependent effects and sGC-independent effects of NO, such as nitr(osyl)ation, and to discriminate between haem-dependent and haem-independent effects of sGC. For example, the role of NO-sGC signalling in the potentially lethal cardiovascular collapse associated with overwhelming systemic inflammation is controversial. Septic or inflammatory shock remains the primary cause of death in intensive care units^16^ and NO signalling is considered a central pathway of the cardiovascular collapse associated with the resulting systemic shock. The prevailing paradigm identifies sGC as a central mediator leading to hypotension, shock and mortality in systemic inflammation^17^,^18^. To test this paradigm, we measured BP, HR and mortality in TNF-induced systemic shock in apo-sGC and wild-type (WT) mice. Our findings that apo-sGC mice are not protected from the cardiovascular collapse and lethality associated with TNF-induced systemic shock, indicate that sGC is not a central mediator of hypotension, shock and mortality in systemic shock.

## Results

### Generation of apo-sGC mice

Apo-sGC mice were generated using standard transgenic methods using a targeting strategy designed so that the WT exon 5 of the endogenous *Gucy1b3* gene was replaced with a mutant exon 5 carrying the H105F point mutation, resulting in replacement of the WT histidine, which is responsible for ligation of the haem-group to sGC^19^, with a phenylalanine (Supplementary Fig. 1a.). Analysis of progeny showed that mice heterozygous (HE) and homozygous knock-in (KI) for the knock-in mutation were viable but with a skewed Mendelian segregation in the offspring of HE breeders at 21 days of life (27% WT (*n*=1,172), 51% HE (*n*=2,252), 22% apo-sGC (*n*=987); *χ*^2^=17.5; *P*<0.001).

### Molecular characterization of apo-sGC mice

**Figure 1:**
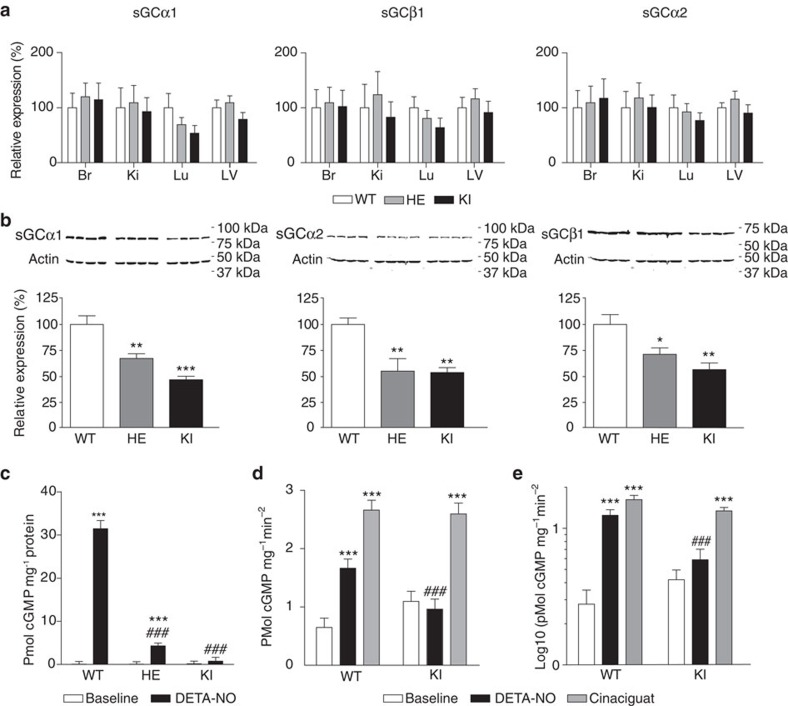
Molecular characterization of apo-sGC mice. (**a**) Quantitative RT–PCR measurement of mRNA encoding sGC subunits in the brain (BR), the kidney (KI), the lung (LU) and left ventricle (LV; *n*=6 for all genotypes). (**b**) Representative and quantification of sGC subunits in brain homogenates of WT, HE and KI mice. *P<0.05, **P<0.01 and ***P<0.001 versus WT by one-way ANOVA (*n*=4 for all genotypes). Data are mean±s.e.m. from three independent experiments. (**c**) Aortic cGMP levels in untreated (WT: *n*=12, HE: *n*=11, KI: *n*=11) and DETA-NO-treated mice (60 mg kg^−1^; WT: *n*=7, HE: *n*=7, KI: *n*=7). ***P<0.001 versus baseline and ^###^*P*<0.001 versus WT by one-way ANOVA. (**d**,**e**) Basal (WT: *n*=20, KI: *n*=17), DETA-NO-stimulated (WT: *n*=20, KI: *n*=17) and cinaciguat-stimulated (WT: *n*=18, KI: *n*=15) sGC enzyme activity in lung (**d**) and aortic (**e**) extracts of WT and KI mice. ***P<0.001 versus baseline, ^#^*P*<0.05 and ^###^*P*<0.001 versus WT by one-way ANOVA.

mRNA expression levels of transcripts for sGCα1, sGCα2 and sGCβ1 protein were similar in the brain, the kidney, the lung and the left ventricle of apo-sGC (KI), HE, and WT mice (Fig. 1a), indicating that introduction of the H105F mutation in sGCβ1 does not affect expression of sGC subunits. Similarly, no changes were observed in the expression of genes encoding other relevant proteins in the NO/cGMP signalling pathway (Supplementary Fig. 2). In contrast to the similar gene expression levels in the different genotypes, sGCα1, sGCα2 and sGCβ1 protein levels were lower in tissues of apo-sGC and HE mice than in WT mice (Fig. 1b and Supplementary Fig. 3). Basal cGMP levels in tissues did not differ between apo-sGC, HE and WT mice (Fig. 1c). Baseline sGC activity was similar in lung and aorta homogenates of apo-sGC and WT mice (Fig. 1d,e). The ability of the NO-donor compound diethylenetriamine NO (DETA-NO) to increase cGMP levels *in vivo* was attenuated and absent in aortas of HE or apo-sGC mice, respectively (Fig. 1c). Similarly, DETA-NO was unable to increase sGC activity in tissue extracts from the lung and the aorta of apo-sGC mice (Fig. 1d,e). In contrast, cinaciguat, a haem-independent sGC activator, activated sGC in apo-sGC tissue extracts (Fig. 1d,e).

### Apo-sGC enzyme function in CHO cells

**Figure 2:**
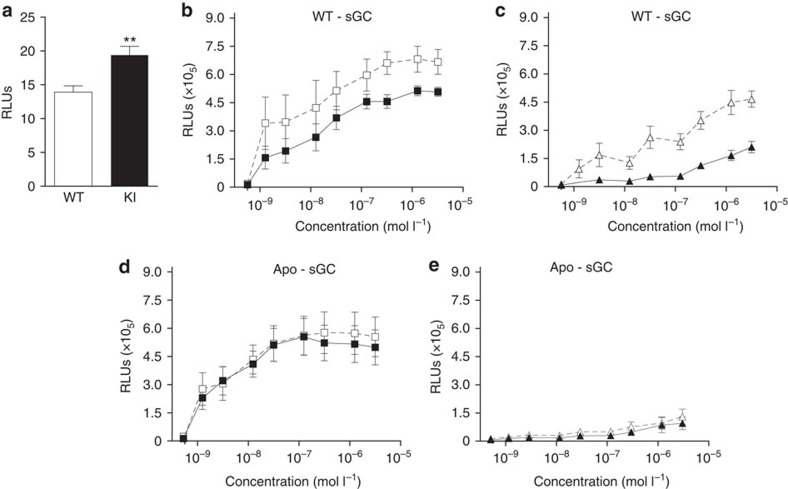
Characterization of WT and apo-sGC in CHO cells. (**a**) cGMP readout cells were co-transfected with WT-sGCα1 in combination with the WT sGCβ1-subunit or the mutant sGCβ1^H105F^-subunit of sGC to generate WT- and apo-sGC, respectively. Baseline activity of WT-sGC (WT) and apo-sGC (KI). **P<0.01; unpaired *t*-test. (**b**–**e**) Activation pattern of WT-sGC (**b**,**c**) and apo-sGC (**d**,**e**) incubated with increasing concentrations of cinaciguat alone (□; **b**,**d**), increasing concentrations of cinaciguat in combination with a fixed concentration of 10 μM ODQ (□; **b**,**d**), increasing concentrations of BAY 41-2272 alone (▴; **c**,**e**), or increasing concentrations of BAY 41-2272 in combination with a fixed concentration of DEA/NO (▵; **c**,**e**). Enzyme activity is represented as relative light units (RLUs). Data are mean±s.e.m. from 5 to 7 independent experiments performed in quadruple, statistical analysis by Student's *t*-test.

The combined observation of reduced sGC protein levels together with preserved basal cGMP levels suggests that the apo-sGC enzyme has a higher basal activity than the WT enzyme. To test this hypothesis, we assessed apo-sGC enzyme function *in vitro* in a cGMP reporter cell line. Basal cGMP production was higher in reporter cells expressing haem-free sGCβ_1_^H105F^ than in cells expressing WT-sGC (Fig. 2a).

In reporter cells expressing WT, haem-containing sGC, cGMP production was increased in a concentration-dependent manner by cinaciguat (Fig. 2b). Adding the selective sGC inhibitor 1H-[1,2,4] oxadiazolo [4,3-a] quinoxalin-1-one (ODQ), which exerts its activity through oxidation of the haem, enhanced the ability of cinaciguat to activate sGC (Fig. 2b). Stimulation with BAY 41-2272 increased cGMP synthesis in a concentration-dependent manner (Fig. 2c). Combination of BAY 41-2272 with 1 nmol l^−1^ diethylamine NONOate (DEA/NO) synergistically stimulated sGC (Fig. 2c).

In reporter cells expressing apo-sGC, cinaciguat increased the activity of apo-sGC (Fig. 2d) in a dose-dependent manner. Addition of 10 μM ODQ did not enhance cinaciguat-induced sGC activity (Fig. 2d). In contrast, neither BAY 41-2272 alone at concentrations previously demonstrated to specifically activate sGC but not inhibit phosphodiesterase (PDE) activity nor BAY 41-2272 in combination with NO was able to activate apo-sGC (Fig. 2e). These data indicate that the activity of apo-sGC is higher than WT sGC at baseline and that apo-sGC can concentration dependently be activated by haem-independent sGC activators but not by haem-dependent sGC stimulators.

### Impaired NO-mediated platelet inhibition in apo-sGC mice

**Figure 3:**
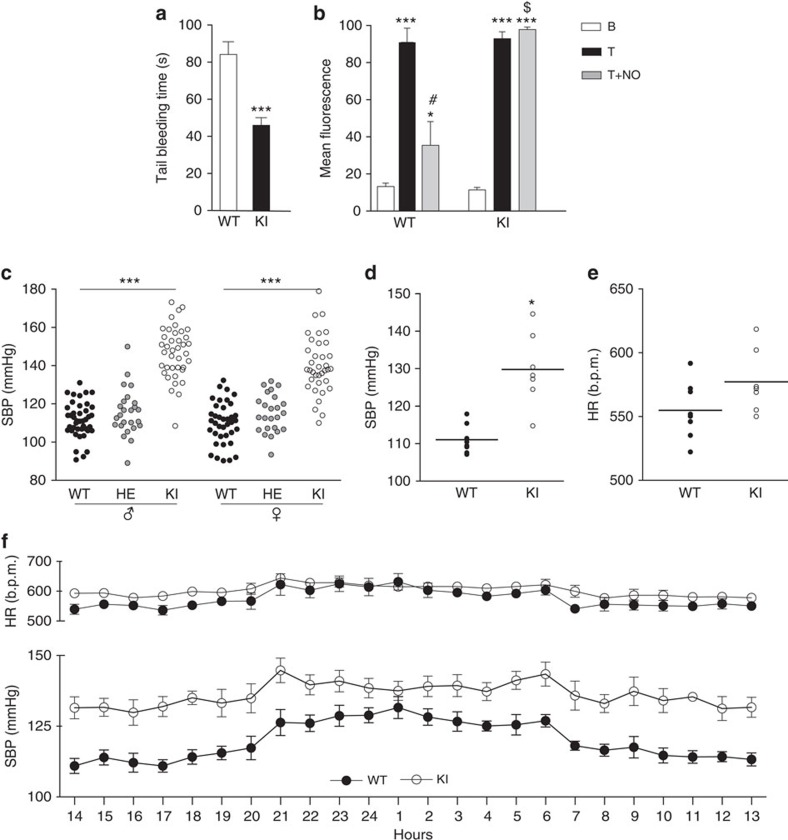
Ablation of NO-inhibited platelet activation and hypertension in apo-sGC (KI) mice. (**a**) Tail bleeding time in WT and KI mice. ***P<0.001 versus WT by one-way ANOVA (WT: *n*=29, HE: *n*=30 and KI: *n*=27). (**b**) Baseline (B), thrombin-induced (T) and NO-inhibited (T+NO) platelet activation by measurement of CD62P-FITC mean fluorescence in platelets of WT and KI mice. *P<0.05 and ***P<0.001 versus baseline, ^#^*P*<0.05 versus thrombin and ^$^*P*<0.05 versus WT thrombin+NO by two-way ANOVA (WT: *n*=4, KI: *n*=4). (**c**) Systolic blood pressure (SBP) measured using tail cuff in conscious male and female WT, HE and KI mice. ***P<0.001 versus WT by one-way ANOVA (male WT: *n*=42, HE: *n*=25 and KI: *n*=39; female WT: *n*=38, HE: *n*=24 and KI: *n*=36). Mean of 1000 hours to 1700 hours telemetric recordings for SBP (**d**) and heart rate (HR) (**e**) in KI and WT mice. *P=0.025 versus WT by Student's *t*-test (WT: *n*=8, KI: *n*=7). (**f**) Representative 24-h telemetric recordings of hourly means±s.e.m. of SBP and HR in WT and KI mice (WT: *n*=8, KI: *n*=7).

Tail bleeding time, an *in vivo* measure of platelet reactivity, was shorter in apo-sGC mice than in WT littermates (Fig. 3a). In addition, platelet activation studies were performed on isolated platelets of WT and apo-sGC mice by measuring the ability of an NO donor compound (sodium nitroprusside (SNP)) to modulate thrombin-induced surface expression of CD62P. Although stimulation of platelets with thrombin resulted in a similar upregulation of CD62P on WT and apo-sGC platelets, SNP inhibited CD62P expression on WT but not apo-sGC platelets (Fig. 3b). Together, these data demonstrate that inhibition of platelet activation is impaired in apo-sGC mice, suggesting that the ability of NO to inhibit platelet activation critically depends on sGC.

### Haemodynamic response to NO is absent in apo-sGC mice

**Figure 4:**
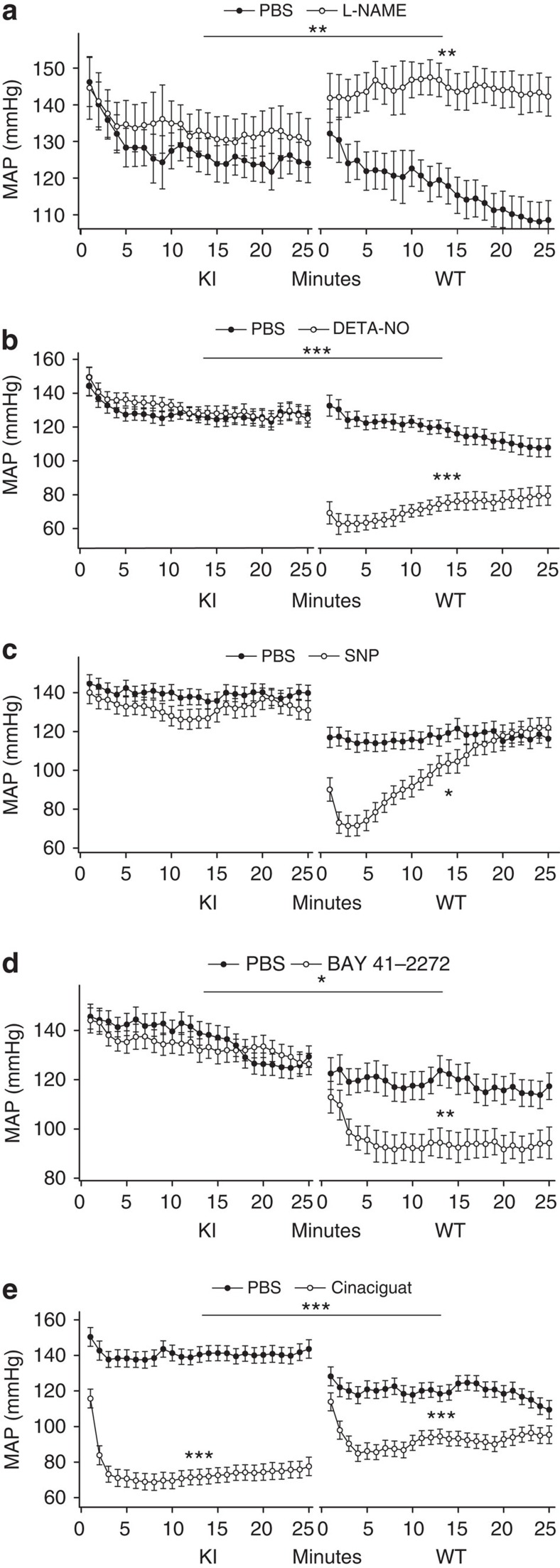
Cinaciguat but not NO or BAY 41-2272 decreases mean arterial pressure (MAP) in apo-sGC (KI) mice. MAP±s.e.m. measured telemetrically in KI and WT mice 1–25 min after challenge with PBS (10 ml kg^−1^ IV; WT: *n*=8, KI: *n*=9; **a**,**b**), L-NAME (100 mg kg^−1^ IV; WT: *n*=7, KI: *n*=4; **a**), DETA-NO (60 mg kg^−1^ IV; WT: *n*=7, KI: *n*=9; **b**), PBS (10 ml kg^−1^ IP; WT: *n*=7, KI: *n*=10; **c**), SNP (1.5 mg kg^−1^ IP; WT: *n*=5, KI: *n*=6; **c**), vehicle (10 ml kg^−1^ IP; WT: *n*=5, KI: *n*=9; **d**), BAY 41-2772 (4 mg kg^−1^ IP; WT: *n*=5, KI: *n*=9; **d**), vehicle (10 ml kg^−1^ IV; WT: *n*=7, KI: *n*=7; **e**) or cinaciguat (300 mg kg^−1^ IV; WT: *n*=8, KI: *n*=7; **e**). *P<0.05, **P<0.01 and *** *P*<0.001 compared with PBS or vehicle by residual maximum likelihood (REML) (see also Supplementary Tables 3–7).

We next investigated the *in vivo* impact of oxidized sGC on cardiovascular function by measuring BP and heart rate (HR) in freely moving conscious WT and apo-sGC mice by telemetry, and in restrained mice by tail-cuff. Systolic BP (SBP) was higher in conscious apo-sGC mice than in WT mice (Fig. 3c,d,f). HR did not differ between apo-sGC and WT mice (Fig. 3e,f). Administration of the NOS inhibitor N^ω^-nitro-L-arginine methyl ester (L-NAME) *in vivo* increased BP and decreased HR in WT but not in apo-sGC mice (Fig. 4a, Supplementary Fig. 4a, 5a,b and Supplementary Table 3). Similarly, *in vivo* administration of the exogenous NO donors DETA-NO, an NO-donor with a half-life of 20 h at 37 °C, and SNP, an NO-donor with a half-life of <2 min at 37 °C, decreased BP and increased HR in WT but not in apo-sGC mice (Fig. 4b,c, Supplementary Fig. 4b,c, 5c–f and Supplementary Tables 4 and 5).

**Figure 5:**
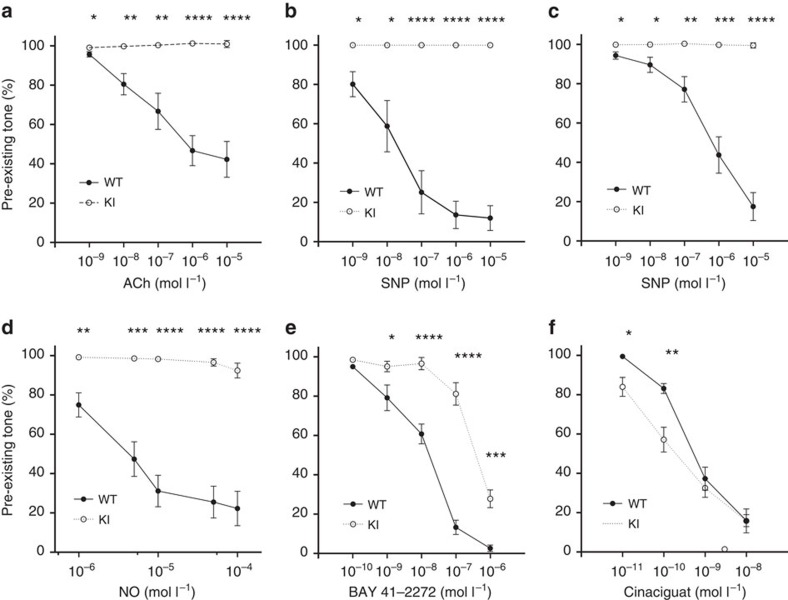
Altered vasoreactivity in apo-sGC (KI) mice. Relaxation effects of acetylcholine (ACh; **a**), sodium nitroprusside (SNP; **b**), *S*-Nitroso-*N*-acetylpenicillamine (SNAP; **c**), NO-gas (**d**), BAY 41-2272 (**e**) and cinaciguat (**f**) on aortas precontracted with 3 × 10^−8^ mg ml^−1^ phenylephrine (PE). *P<0.05, **P<0.01 and ***P<0.001 versus WT by Student's *t*-test (WT: *n*=6, KI: *n*=6 for Ach, SNP, SNAP, NO and WT: *n*=6, KI: *n*=7 for BAY 41-2272 and cinaciguat, respectively).

To further assess how the presence of apo-sGC affects the ability of NO to induce vasorelaxation, we performed vessel reactivity studies on aortic rings isolated from WT and apo-sGC mice. In accordance with the impaired cardiovascular effects of NO in apo-sGC mice *in vivo*, acetylcholine, SNP, *S*-Nitroso-*N*-acetylpenicillamine and NO gas-dependent relaxation were abolished in apo-sGC aortic rings (Fig. 5a–d). Taken together, these data demonstrate that NO-responsive sGC is required for NO to induce vasorelaxation and lower BP.

### Haemodynamic effects of sGC stimulators versus activators

We then addressed whether loss of sGC haem affects the ability of BAY 41-2272 to modulate BP and HR. *In vivo* administration of BAY 41-2272 was associated with a BP decrease in WT mice but not in apo-sGC mice (Fig. 4d, Supplementary Fig 4d, 6c,d and Supplementary Tables 6 and 7). Similarly, the vasorelaxing effect of BAY 41-2272 on WT isolated aortic rings was greatly attenuated in aortic rings of apo-sGC mice (IC_50_≈35 nmol l^−1^ and 1112, nmol l^−1^, respectively; Fig. 5e).

In contrast, *in vivo* administration of cinaciguat decreased BP and increased HR in both apo-sGC mice and WT mice (Fig. 4e, Supplementary Fig. 4e, 6e–f and Supplementary Tables 6 and 7). In fact, the BP-lowering effect of cinaciguat in apo-sGC mice was significantly greater (Fig. 4e) and longer lasting (Supplementary Fig. 7) than in WT mice. In addition, cinaciguat decreased BP in apo-sGC mice at concentrations that did not affect BP in WT mice (Supplementary Fig. 7). Furthermore, the IC_50_ values for cinaciguat-induced *ex vivo* relaxation of precontracted aortas were threefold lower in apo-sGC mice than in WT mice (IC_50_≈0.2 nmol l^−1^ and 0.7 nmol l^−1^, respectively; Fig. 5f). Together, our results suggest that sGC activators like cinaciguat but not sGC stimulators like BAY 41-2272 activate apo-sGC *in vivo*. In addition, the observation that cinaciguat can modulate vasorelaxation and BP in WT mice suggests that even in healthy mice, a subset of the available sGC pool is haem-free and responsive to sGC activators.

### Reduced lifespan and altered morphology of apo-sGC mice

**Figure 6:**
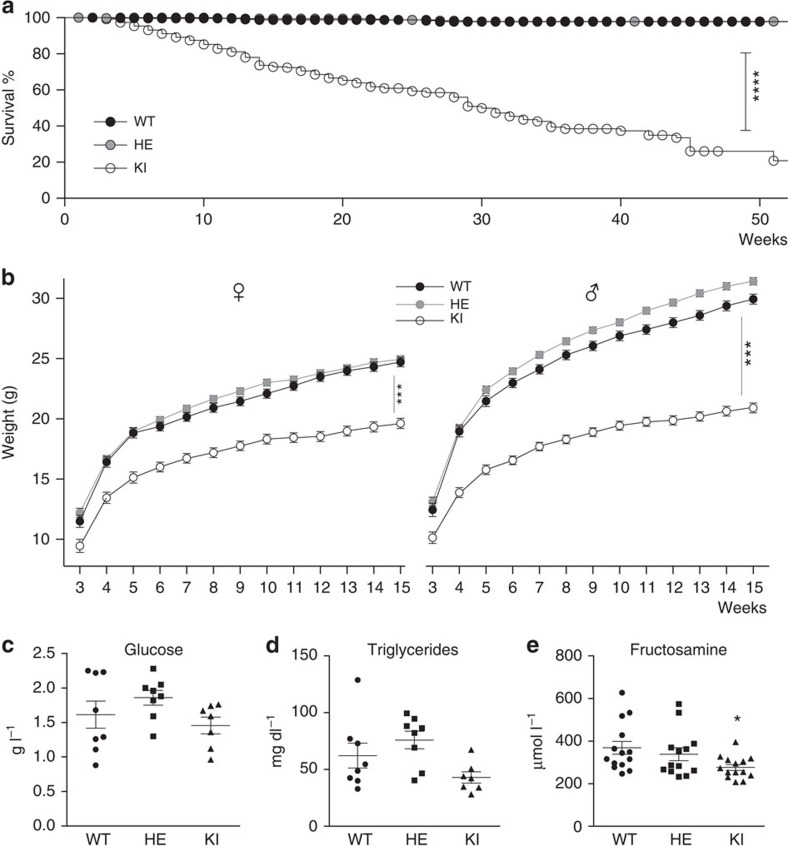
Reduced longevity and growth retardation in apo-sGC (KI) mice compared with WT mice. Survival plots for WT, HE and KI mice (**a**). ***P<0.0001 KI versus WT by Mantel–Cox test (WT: *n*=633, HE: *n*=1370, KI: *n*=545). Body weight versus age in male and female mice (**b**). ***P<0.001 versus WT by residual maximum likelihood (REML) autoregressive order 2 analysis followed by F-test (male WT: *n*=56, HE: *n*=129, KI: *n*=69 and female WT: *n*=63, HE: *n*=121, KI: *n*=52). Scatter plot with mean daily metabolic recordings measured in male apo-sGC (KI) mice and littermate controls expressed per gram of body weight per day±s.e.m. Apo-sGC and HE mice have similar serum glucose (**c**) and triglyceride (**d**) levels as WT mice but have reduced fructosamine (**e**) levels. *P<0.05; one-way ANOVA followed by Dunnett's multiple comparison test (serum glucose and triglycerides: WT: *n*=8, HE: *n*=8 and KI: *n*=7, respectively; fructosamine: WT: *n*=14, HE: *n*=13 and KI: *n*=14, respectively).

Apo-sGC mice are viable but have a reduced lifespan, and display gastrointestinal (GI) abnormalities and growth retardation. Other strains of mice deficient in key enzymes in the NO/sGC/cGMP pathway have a shorter lifespan than WT mice^20^,^21^,^22^. Similarly, we observed a reduced lifespan in apo-sGC mice compared with WT and HE littermates (Fig. 6a). In addition, weight and size of apo-sGC were consistently smaller than that of WT littermates (Fig. 6b). Anatomical observation of apo-sGC mice revealed reduced fat mass (Supplementary Fig. 8a–c), profound gastric enlargement (Supplementary Fig 8a–c), signs of pyloric constriction and an enlarged and translocated caecum (Supplementary Fig. 8a). We assessed for malnutrition or lack of appetite in apo-sGC mice by monitoring food intake (Supplementary Fig. 9a), production of faeces (Supplementary Fig. 9b) and serum levels of fructosamine, glucose and triglycerides (measures of metabolic function). Intriguingly, growth retardation in apo-sGC mice occurred despite a significantly increased food intake (Supplementary Fig. 9a). Serum levels of fructosamine (Fig. 6e), but not glucose or triglycerides (Fig. 6c,d), were lower in apo-sGC mice than in WT mice. These results suggest that growth retardation in apo-sGC mice is not due to malnutrition or malabsorption.

### Apo-sGC mice are not protected against systemic TNF shock

**Figure 7:**
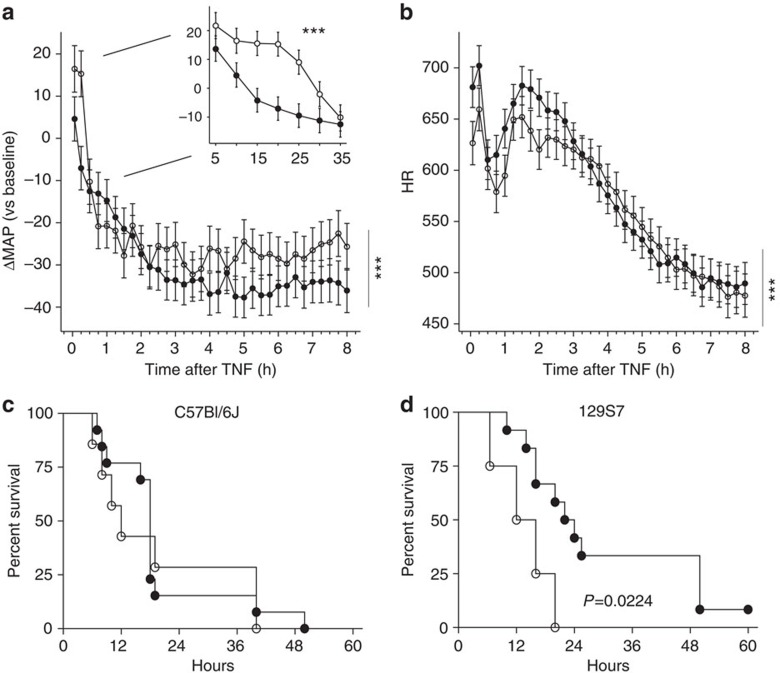
Hypotension and mortality in apo-sGC (KI) mice after TNF-induced systemic shock. Change in MAP (**a**) and HR (**b**) ±s.e.m. measured telemetrically in KI (○) and WT mice (●) 0–8 h after challenge with an LD100 of murine TNF IV (480 μg kg^−1^). The insert in **a** represents the response in MAP during the first 30 min after TNF challenge. ***P<0.001: changes in MAP after challenge compared with control by residual maximum likelihood (REML) (WT: *n*=8, KI: *n*=7, see also Supplementary Tables 8 and 9 and Supplementary Fig. 10 for actual MAP). Survival plots for WT and KI mice after challenge with an LD100 of murine TNF IV (480 μg kg^−1^) on a C57Bl/6J (**c**) or 129S7 (**d**) genetic background. *P* for KI versus WT by Mantel–Cox test.

Administration of a dose of TNF, previously determined to be lethal for WT mice, induced hypotension (Fig. 7a and Supplementary Fig.10) and bradycardia (Fig. 7b) in both WT and apo-sGC mice. During the first 30 min after TNF administration, the decrease in BP was less pronounced in apo-sGC mice than in WT mice (Fig. 7a and Supplementary Fig. 10, insert). The TNF-induced mortality was similar in WT and apo-sGC mice in a C57BL/6J background, and even increased in apo-sGC mice in a 129S7 background (Fig. 7c,d). Together, these findings demonstrate that NO-induced sGC activity is not required for the cardiovascular collapse and lethality associated with TNF-induced inflammatory shock.

## Discussion

Oxidative stress, a major risk factor for cardiovascular disease^1^,^2^,^3^, directly impairs sGC mediated signalling through oxidation of its prosthetic haem group, resulting in the formation of haem-free sGC or apo-sGC. Apo-sGC is insensitive to activation by NO and susceptible to active degradation through the ubiquitin-proteasome pathway^23^. sGC activators such as cinaciguat were designed to specifically target and activate apo-sGC^4^,^7^,^12^ and protect it from degradation^23^. As apo-sGC is a consequence of oxidative stress, it is proposed that targeting apo-sGC and not native sGC, may specifically target diseased organs without having unwanted side effects because of ubiquitous sGC activation. Maximizing the therapeutic potential of cinaciguat and other haem-independent sGC activators requires a thorough understanding of their target, apo-sGC, on a molecular and physiological level, and of their mode of action both *in vitro* and *in vivo*. However, investigations into the specific pathological consequences of impaired sGC activity through haem-oxidation, as well as the consequences of specifically activating the oxidized/haem-free pool of sGC have been hampered by the incapacity to distinguish native, haem-containing sGC from oxidized/haem-free sGC *in vivo*^7^,^8^. We report the generation and cardiovascular characterization of a viable line of apo-sGC mice, expressing a NO-resistant sGC that can be activated by haem-independent sGC activators, and identify novel aspects of the physiological role of sGC and the *in vivo* actions of sGC activators.

The observation that protein levels of sGC subunits are lower in tissues from apo-sGC than in WT mice confirms recent *in vitro* and *ex vivo* findings suggesting that the ferrous haem group protects sGC from degradation^9^,^23^. Nonetheless, the finding that apo-sGC can exist *in vivo* illustrates that targeting oxidized sGC represents a feasible therapeutic approach in cardiovascular (and other) disorders characterized by increased oxidative stress and subsequent oxidation of sGC.

Tissue cGMP levels and sGC catalytic activity in tissue homogenates were similar in WT, HE and apo-sGC mice. When taking into account the lower sGC protein levels in apo-sGC mice than in WT mice, these results suggest that apo-sGC has a higher basal activity than the WT enzyme. Using a cGMP-reporter cell line, we confirmed the higher basal activity of overexpressed apo-sGC *in vitro*. These results support previously reported findings that basal activity of a haem-free mutant of sGC or of detergent-treated haem-free sGC that lacks the characteristic Soret peak at 431 nm is higher than that of WT sGC^24^,^9^. Together, these findings suggest that the ligand-bound haem-group acts as an endogenous inhibitor of sGC^25^.

The lifespan of apo-sGC mice is shorter than that of WT mice but similar to that described for mice deficient in all three NOS isoforms^22^. These data suggest that NO affects longevity in an sGC-dependent manner. Importantly, the lifespan of apo-sGC mice, with more than 90% survival at 31 days, is substantially longer than that of sGCβ1^−/−^ mice, with less than 10% of sGCβ1^−/−^ offspring surviving longer than 31 days^20^. The difference in survival between apo-sGC mice and sGCβ1^−/−^ mice suggests a pivotal role for NO-independent sGC activity in determining survival and longevity in mice.

NO-sGC-cGMP signalling was previously demonstrated to be an important mediator in the relaxation of the stomach in reflex to increases in gastric pressure^26^. Similar to observations made in NOS1^−/−^ and cGMP-dependent protein kinase 1^−/−^ (PKGI^−/−^) mice^21^,^27^,^28^, gastric enlargement, signs of pyloric constriction and a delayed GI transit time were present in apo-sGC mice^29^. The gastric enlargement in apo-sGC is therefore likely caused by pyloric constriction and delayed gastric emptying^29^. Comparable to sGCβ1^−/−^ mice^20^, an enlarged and translocated caecum was observed in apo-sGC mice. Together, these data confirm that activation of sGC by NO is necessary for normal GI function and that basal levels of cGMP produced by apo-sGC are not sufficient to fully preserve GI function and morphology. However, basal cGMP production in apo-sGC mice seems sufficient to prevent the fatal GI dysfunction observed in sGCβ1^−/−^ mice^20^. It is conceivable that the observed growth retardation is secondary to abnormal GI function. However, normal triglyceride and glucose levels suggest that impaired GI function in apo-sGC mice does not result in malnutrition. It remains to be determined whether alterations in metabolism or tissue development contribute to the reduced fat mass observed in apo-sGC mice despite an increase in food uptake.

The ability of NO to inhibit platelet activation^30^, to induce vascular smooth muscle relaxation and to lower BP is well characterized^31^. Consistent with results in sGCβ1^−/−^, sGCα1^−/−^ and PKGI^−/−^ mice^20^,^32^,^33^,^34^, lack of NO-dependent sGC activation completely abrogated the anti-platelet and BP-lowering effects of NO. These results demonstrate that haem-containing, reduced sGC is required for NO's effects on platelets and systemic vasorelaxation.

sGC stimulators are a novel class of molecules that stimulate the catalytic activity of sGC, and that have been successfully developed for therapeutic use in PAH^13^. They are dependent on interaction with the sGC haem-group to activate the enzyme, and can activate sGC in a synergistic manner with NO^35^. The ability of BAY 41-2272 to activate apo-sGC, either alone or in synergy with NO, or to relax aortic rings was severely attenuated. The residual effect of BAY 41-2272 in aortic rings of apo-sGC mice is likely due to off target effects, including its inhibitory effect on phosphodiesterases at concentrations higher than 5 μmol l^−1^ (refs 35, 36). Similarly, administration of BAY 41-2272 decreased BP in WT but not apo-sGC mice. These results confirm the absence of haem in the apo-sGC enzyme and demonstrate that apo-sGC cannot be activated by BAY 41-2272 *in vivo*.

Cinaciguat, a member of a new class of haem-independent sGC activators, exclusively activates haem-free sGC by binding in the haem pocket and changing the enzyme's conformation^14^. In a cGMP reporter cell line transfected with WT sGC, ODQ, a known oxidizer and inhibitor of sGC, potentiated the ability of cinaciguat to activate sGC. In contrast, ODQ did not affect the activation profile of cinaciguat in a cGMP reporter cell line transfected with apo-sGC, indicating again that apo-sGC is completely haem-free. It is noteworthy that, despite lower sGC protein levels in tissues isolated from apo-sGC mice than WT mice, IC_50_ values for cinaciguat-induced *ex vivo* relaxation of precontracted apo-sGC aortas were lower than those in WT aortas. In addition, cinaciguat had a more potent effect on BP in apo-sGC mice than in WT mice. Our *ex vivo* aortic ring experiments and *in vivo* BP observations validate the hypothesis that cinaciguat can activate haem-free/oxidized sGC in relevant tissues.

The observation that cinaciguat also has effects on sGC activation, vasorelaxation and BP in WT cellular systems, WT organ baths and WT mice, respectively, is an indication that in normal circumstances, a subset of the available pool of sGC is haem-free. Consequently, sGC activators may also impact healthy tissue. Whether this is due to the presence of low levels of oxidative stress in healthy organisms or whether the interaction of the haem group with sGC is dynamic *in vivo* remains unresolved. In the latter case, at higher concentrations, cinaciguat might be able to compete with the haem group for binding to sGC as was suggested previously^8^. When attempting to estimate the amount of apo-sGC in healthy tissues, it should be stressed that the correlation between sGC activity, the amount of cGMP produced and the resulting physiological response is weak. For example, in sGCα1^−/−^ mice, the sGCα2β1 isoform, expressed at 20-fold lower levels than sGCα1β1 in vascular tissue, was sufficient to mediate a robust vasorelaxing response and BP lowering effect of NO, in the absence of a measurable increase in cGMP content^32^,^33^. More importantly, by titrating the dose of cinaciguat *in vivo*, we were able to observe a reasonable therapeutic window in which beneficial properties of reactivation of haem-oxidized sGC might be achieved, while avoiding unwanted systemic side effects. Our observations imply that careful dosing and titration might be necessary in developing therapeutic approaches to attain specificity for diseased organs.

Together, these results demonstrate the validity of the concept that loss of the haem of sGC *in vivo*, suggested to occur during conditions of increased oxidative stress^4^, results in elevated systemic BP that can be decreased by sGC activators such as cinaciguat. sGC presents advantages as a therapeutic target for pathologies associated with oxidative stress for two reasons. First, it is downstream from NOS enzymes, which were shown to exert other functions besides sGC activation and which produce free radicals themselves under conditions of oxidative stress. Second, sGC is a target of oxidative damage and can be reactivated by haem-independent sGC activators like cinaciguat. The apo-sGC mouse model provides a unique experimental platform to directly study both the effects of the increased presence of oxidized or haem-free sGC, and the therapeutic ability of sGC activators.

Mortality associated with systemic inflammatory response syndromes (SIRS), such as the cytokine storm syndrome and sepsis, is the highest in the subset of patients who develop shock. The pathophysiology of the first stages of SIRS, including infection, and immune and inflammatory reactions, is quite heterogeneous, with mechanisms ranging from immune paralysis to cytokine storm^37^. However, the development of the cardiovascular collapse in SIRS has generally been considered to depend on a few common mechanisms, of which NO-sGC-cGMP signalling is a major culprit in vasodilation, distributive shock and cardiodepression^17^,^18^. In both animal models and patients, NO produced by inducible NOS2 was implicated in both vasodilation and cardiodepression during shock. Unfortunately, in subsequent clinical trials, inhibition of NOS was found to increase BP in sepsis patients but failed to improve survival. In fact, treatment with non-isoform-specific NOS inhibitors was associated with higher mortality^38^. Further research identified the existence of NOS-dependent protective cardiovascular effects in SIRS. We previously reported that mice deficient in sGCα_1_ (sGCα_1_^−/−^ mice) are sensitized to the cardiac dysfunction and mortality associated with inflammatory shock (both endotoxin-induced and TNF-induced shock), suggesting that sGCα_1_β_1_-derived cGMP is cardioprotective in murine inflammatory shock models. In addition, we have recently shown that the haem- and NO-independent sGC activator BAY 58-2667 (Cinaciguat), can improve organ function and survival during lethal endotoxemia when given in the appropriate treatment window^39^. Together, these considerations frame the ongoing debate on the exact role, be it detrimental^40^,^41^,^42^,^43^,^44^ or protective^45^,^46^,^47^,^48^, of NO-sGC-cGMP signalling in the cardiovascular collapse during shock.

The availability of NO-insensitive apo-sGC mice facilitates research into testing the hypothesis whether the cardiovascular collapse in SIRS depends on NO-dependent sGC activation. To do so, we opted for a TNF-induced SIRS model characterized by a marked increase in systemic NO levels, in which the cardiovascular collapse is a most prominent feature, and which mimics the human situation in its cardiovascular aspects^49^,^50^,^51^. Using a downstream effector as a challenge instead of an infectious challenge avoids as much as possible that the observed consequences of modulation of the NO-sGC-cGMP pathway would be due to interference with the effects of this pathway on infection control and on the regulation of the generation of an immune and inflammatory response itself, rather than on the effects of NO-sGC-cGMP on the cardiovascular system. Indeed, NO-sGC-cGMP signalling has also effects outside the cardiovascular system, such as in infection control and in the regulation of the generation of an immune and inflammatory response. Administration of a lethal dose of TNF induced similar hypotension and bradycardia in WT and apo-sGC mice. Only in the first 30 min after TNF administration, a time at which NO is derived from NOS3 rather than NOS2, the decrease in BP was more pronounced in WT than in apo-sGC mice. This suggests that the early, NOS3-derived effect of TNF on BP is mediated in part by the sGC/cGMP pathway, whereas the later effects are sGC independent. The TNF-induced mortality was similar in WT and apo-sGC mice on a C57BL/6J background, and even higher in apo-sGC mice than in WT mice on a 129S7 background. Even though a poor nutritional status has been associated with increased mortality in inflammatory shock^52^, it is unlikely that malnutrition contributes to the increased mortality in apo-sGC mice taking in to account the normal triglyceride and glucose levels of apo-sGC mice. These results indicate that NO-sGC-dependent signalling is not required for cardiovascular collapse or for mortality associated with inflammatory shock. Together with recent observations that excessive production of systemic NOS2-derived NO is not necessarily associated with haemodynamic collapse^53^, our observations in apo-sGC mice challenge the prevailing paradigm of the central and crucial role of NO/sGC signalling in inflammatory shock. An alternative mechanism leading to septic shock might be the direct oxidative activation of PKG1 as proposed recently^2^. These observations do not exclude protective or deleterious involvement of NO-sGC-dependent signalling in the antimicrobial or inflammatory aspects of the pathophysiology of sepsis but only imply that NO-sGC signalling is not the pathway resulting in cardiovascular collapse.

In conclusion, this paper describes the cardiovascular phenotype of a novel mouse model that allows us to distinguish between sGC-dependent and sGC-independent effects of NO and between haem-dependent and haem-independent effects of sGC. Our results highlight the importance of sGC as the primary receptor for NO in cardiovascular physiology, and identify an important role for haem-independent activity of sGC in determining survival and longevity. In addition, we demonstrated that, in a model of TNF-induced systemic shock, NO/sGC/cGMP signalling is not responsible for the prolonged refractory hypotension or for mortality. The data presented here illustrate the therapeutic potential of compounds able to activate apo-sGC in setting of cardiovascular disease, including systemic hypertension, providing a solid scientific ground for the further development of this new class of drugs.

## Methods

### Generation of apo-sGC mice

The sGCβ1-targeting vector was constructed using a 10.5-kb genomic DNA contig picked up by screening of a 129SvJ lambda FIX II mouse genomic library (Stratagene). A targeting vector containing a 3-kb 3′ and a 4.1-kb 5′ flanking homology, a positive, *loxP* flanked (neomycin-resistance cassette, *Neo*^*r*^) and a negative (thymidine kinase cassette, *TK*) selection marker was constructed, in which the histidine residue at position 105, located in exon 5, was mutated to phenylalanine (Supplementary Fig. 1). Five other silent mutations were introduced as well. The mutations were introduced using the Transformer Site-Directed Mutagenesis Kit (Clontech Laboratories, Inc.). Targeting was applied in a 129SvEv mouse embryonic stem cell line^54^ and 263 G418-resistant clones were selected from the respective lines. Correct homologous recombination was confirmed through Southern blot (Supplementary Fig. 1b, c) and multiple integration events were ruled out by PCR (Supplementary Fig. 1d). One positive clone was expanded and subjected to Cre-mediated deletion of the neomycine cassette by transient transfection with the Cre-expression plasmid pCAGGS-Cre. G418-sensitive clones were selected and screened for successful deletion of the neo cassette through Southern blot (Supplementary Fig. 1b, c) and PCR (Supplementary Fig. 1d). *In vitro* Cre-Lox-mediated deletion of the *Neo*^*r*^-cassette after transfection of 129SvEv embryonic stem cells was verified by Southern blot using probes located at the 5′ and 3′ end of the construct, and by PCR (primers: P1F: 5′- GACCCACAGCCCAAGTGTAAC-3′ ; P1R: 5′- GAAGAGCTTGGCGGCGAATG-3′ ) spanning the entire 3 kb homology arm. Chimeras were generated by blastocyst injection of C57Bl/6J blastocyst-stage embryos with a correctly targeted ES cell clone^54^ and implantation in a pseudo-pregnant female. Germline transmission was verified by mating male chimeras to C57Bl/6J females. Subsequent generation of apo-sGC mice by mating male and female mice HE for the mutated allele, was confirmed by Southern blot and RT–PCR (Supplementary Fig. 1e; primers: P2F: 5′- GAACATTAAATTAGCCG-3′ ; P2R: 5′- GTTATCAATAGCu8ATCAGG-3′ ).

### Animals

Mice were bred and maintained in specific pathogen-free conditions in the animal facility of IRC/VIB1, Ghent. Mice were housed in temperature- and humidity-controlled conditions at 21 °C and 40–65% relative humidity, 14 h/10 h light/darkness, with *ad libitum* food and water. Mice were fed irradiated ‘Transbreed' chow (SDS), unless stated otherwise, and regular drinking water. The genetic background of the mice is an F2 mixed background of 129SvEv x C57Bl/6J, except if stated otherwise. In all experiments, equal numbers of male and female mice between 12 and 18 weeks were used, unless stated otherwise. In all experiments, littermates were used as controls. All experiments were conform to the Belgian and European law on laboratory animal experimentation and were approved by the Local Ethical Committee of Ghent University.

### Growth curve

Apo-sGC mice were fed three different rodent chows: standard pellets (SNIFF), expanded diets (RM3, SDS) and a high-energy and high-nutrient diet (Transbreed, SDS). Body weights were measured from 3 to 15 weeks of age. As no statistical interaction was observed between growth curves on different diets, all curves were combined in one final growth curve.

### Metabolic cages

Mice were placed individually in metabolic cages (Techniplast) with food and water *ad libitum*, and allowed to adapt for 3 days. Starting from day 4, 24 h faeces were collected and 24 h food uptake was measured for 3 consecutive days.

### Collection and analysis of blood

Mice were fasted for 16 h, anaesthetized with isoflurane and blood was collected by cardiac puncture and kept for 30 min at room temperature to promote clot formation. Serum was prepared by cold centrifugation at 3000*g* for 20 min. Serum was stored at −20° C until use. Serum glucose was determined using routine methods using commercial reagents (Roche) on a Cobas 6000 analyzer (Roche). Fructosamine levels were determined on a Cobas-Mira Chemistry Analyzer using the Roche reagents (Roche). Triglyceride levels were determined with the EnzyChrom Triglyceride Assay Kit (BioAssay Systems) according to the manufacturer's instructions.

### Harvesting of organs

Mice were killed by cervical dislocation and organs were quickly dissected and weighed or snap frozen for immunoblot, sGC activity, cGMP concentration and RT Q–PCR, and stored at −70 °C.

### Immunoblot analysis and quantification of sGC subunits

Tissue samples were pulverized at −70 °C (Retsch MM301), homogenized in 1 ml ice-cold lysis-buffer (HEPES 20 mmol l^−1^, NaCl 350 mmol l^−1^, Triton X-100 0.5%, EDTA 0.5 mmol l^−1^, Glycerol 20%) supplemented with 1 μmol l^−1^Pefabloc SC (Roche Diagnostics), 0.3 μmol l^−1^ Aprotinin (Roche Diagnostics), 1 μmol l^−1^ Leupeptin (Roche Diagnostics), 25 mmol l^−1^ β-glycerolphosphate, 1 mmol l^−1^ NaOVO_3_, 5 mmol l^−1^ NaF and ‘complete' EDTA-free protease inhibitor cocktail (Roche), using a mechanical homogenizer (Polytron PT1600E) for protein extraction. Samples were centrifuged at 14,000*g* for 20 min at 4 °C. Protein extracts (brain 100 μg, lung 100 μg) were separated on a 10% SDS–polyacrylamide gel and transferred to nitrocellulose filters. sGCα1 and sGCβ1 were detected using a rabbit anti-rat antibody from Sigma (G4280 and G4405, respectively, 1:500), and for sGCα2, a rabbit anti-human antibody from Abcam (ab42108 1:500) was used. Immunoblotting with monoclonal mouse anti-human actin (MP Biomedicals, 1:2000) was performed to correct for equal loading. Detection and analysis were performed with the Odyssey system (Odyssey 2.1.12, Li-Cor Biosciences) using fluorophore-coupled secondary antibodies (goat anti-mouse IRDye 680, goat anti-rabbit IRDye 800CW, Li-Cor Biosciences, all at 1:5,000). Results were normalized to actin and expressed as a percentage of WT.

### sGC enzyme activity

To measure sGC enzyme activity, lung, aorta, brain and left ventricle tissues were harvested and homogenized in buffer containing 50 mmol l^−1^ Tris-HCl (pH 7.6), 1 mmol l^−1^ EDTA, 1 mmol l^−1^ dithiothreitol and 2 mmol l^−1^ phenylmethyl sulphonyl fluoride. Extracts were centrifuged at 20,000*g* for 20 min at 4 °C. Supernatants (containing 50 μg protein) were incubated for 10 min at 37 °C in a reaction mixture containing 50 mmol l^−1^ Tris-HCl (pH 7.5), 4 mmol l^−1^ MgCl_2_, 0.5 mmol l^−1^ 1-methyl-3-isobutylxanthine, 7.5 mmol l^−1^ creatine phosphate, 0.2 mg ml^−1^ creatine phophokinase, 1 mmol l^−1^ L-NAME and 1 mmol l^−1^ GTP with or without 10 μmol l^−1^ DETA-NO, 100 μmol l^−1^ cinaciguat or 1 mmol l^−1^ Bay 41-2272. The reaction was terminated by adding 0.9 ml of 0.05 mol l^−1^ HCl. cGMP was measured using a commercial radioimmunoassay (Biomedical Technologies). sGC enzyme activity is expressed as picomoles of cGMP produced per minute per milligram of protein in extract supernatant.

### cGMP concentrations

Mice were dissected immediately or 10 min after intraperitoneally (IP) injection of 60 mg kg^−1^ DETA-NO. Harvested organs were first powdered at −70 °C (Retsch MM301) and subsequently homogenized in 1 ml ice-cold 100% ethanol. Extracts were centrifuged at 14,000*g* for 10 min at 37 °C. The supernatant was transferred and the pellet was washed once with 0.5 ml ice-cold 100% ethanol and centrifuged at 14,000*g* for 10 min at 37 °C. The total supernatant was dried under vacuum at 30 °C. The pellet was redissolved in protein buffer (20 mmol l^−1^ HEPES, 350 mmol l^−1^ NaCl, 0.5 mmol l^−1^ EDTA, 20% glycerol, 0.5% Triton X-100, EDTA-free protease inhibitor mix), centrifuged for 10 min at 14,000*g* at 37 °C and protein concentration of the supernatant was measured using a BCA Protein Assay Kit (Pierce). cGMP pellets were dissolved in EIA-buffer and cGMP concentrations were measured using the acetylation-protocol, of the Cyclic GMP EIA Kit (Cayman Chemical). cGMP concentration is expressed as picomole cGMP per milligram of protein.

### Measurement of mRNA expression levels

Tissues were harvested, rinsed in PBS, snap frozen in liquid N_2_ and stored at −70 °C until RNA was extracted using the RNeasy Plus Protect Mini kit from QIAGEN according to the manufacturer's instructions. One μicrogram of RNA was used in the iScript cDNA Synthesis Kit (Bio-Rad) for RT–PCR to produce cDNA using random hexameric primers. cDNA was subsequently used for relative expression quantification using the LightCycler 480 SYBR Green I Master mix (Roche Diagnostics) in a Lightcycler 480 (Roche). To this end, subunit-specific primer sets were synthesized (see Supplementary Table 1). To normalize the data, the geometric mean of the two most stable housekeeping genes, 60S ribosomal protein L13 (*RPL13*) and β-actin (*ACTB*), out of five tested genes, calculated with the geNorm Housekeeping Gene Selection Kit (PrimerDesign Ltd) was used^55^. Each sample was measured in triplicate to determine the threshold cycle (Ct). For each sample, the normalization factor was calculated as the difference between the geometric mean Ct of the housekeeping genes of the sample and the mean Ct of the housekeeping genes of all samples. The level of target mRNA, relative to the mean of the reference housekeeping genes, was calculated by raising 2 to the power of [40 - (Ct of target - housekeeping gene normalization factor)]. Data±s.e. are plotted as relative amount versus WT male=100%.

### Characterization of WT and apo-sGC in a CHO reporter cell line

Mutagenesis of a plasmid expressing WT sGCβ1 was performed using the QuikChange site-directed mutagenesis kit according to the manufacturer's protocol. The following primer was used to perform the desired mutation: β1^H105F^:5′- CGATGCCCTGTTCGACCACCTCG -3′. The accuracy of the mutation was verified by sequencing (Invitek).

To characterize the sGC mutant in an intracellular environment, a cGMP reporter cell was constructed based on a method reported earlier^56^. Briefly, a CHO cell line expressing cytosolic aequorin was stably transfected with a plasmid coding for the cGMP-gated ion channel CNG2 under a zeocin resistance. Thereafter, zeocin-resistant clones were characterized for a channel expression, and active clones were subcloned by the limited dilution technique. Selected clones were cultured in Dulbecco's modified Eagle's medium pyruvate /F12 with L-glutamine (Invitrogen), 1 nmol l^−1^ sodium pyruvate and 50 μg ml^−1^ streptomycin, 2.5 μg ml^−1^ amphotericin B and 10% (v/v) inactivated fetal calf serum.

For cGMP readout cells were seeded on 96-well microtitre plates at a density of 10,000 cells per well and were cultured for 1 day at 37 °C and 5% CO_2_ to ensure confluent growth. Afterward, cells were co-transfected applying a transfection mixture containing 9 ng of α1- and 9 ng of β1-plasmid, 0.12 μl of Plus reagent and 0.75 μl of LipofectAMINE (Invitrogen) in 100 μl OptiMEM serum-free medium (Invitrogen) according to the manufacturer's protocol. After 3 h at 37 °C, the transfection medium was replaced with serum-containing medium and cells were incubated for 24 h at 37 °C to ensure optimal expression of WT- and mutant-sGC.

Cells were cultured and transiently transfected with WT-sGC or mutant-sGC as described above. 24 h after transfection medium was removed, and the transfected cells were incubated with calcium-free buffer ((130 nmol l^−1^ NaCl, 5 mmol l^−1^ KCl, 20 mmol l^−1^ HEPES, 1 nmol l^−1^ MgCl_2_, 4.8 nmol l^−1^ NaHCO_3_, pH 7,4) containing 0.83 μg ml^−1^ coelenterazine for 3 h at 37 °C. For the determination of the sGC activation profile, cells were incubated with various concentrations of sGC activator, cinaciguat or sGC stimulator, BAY 41-2272 or in the presence of 10 μmol l^−1^ ODQ or 1 nmol l^−1^ DEA/NO in a volume of 50 μl for 10 min at RT. 3-Isobutyl-1-methylxanthin was used in a final concentration of 66.7 μM to prevent cGMP degradation by endogenous PDEs. cGMP readout was initiated by application of buffer containing 10 mmol l^−1^ CaCl_2_ and emitted light was measured as relative light units in a light-tight box using charge-coupled device camera.

### Platelet activation

To isolate platelets, 400 μl blood was collected in 100 μl Acid Citrate Dextrose buffer (16.77 g l^−1^ tri-sodium citrate dihydrate; 14 g l^−1^, citric acid; 18.016 g l^−1^, dextrose; pH 6.5) and diluted in 500 μl PIPES-buffered saline. Platelet-rich plasma was prepared by centrifugation of samples at 100*g* for 20 min. To prepare platelets, the supernatant of the platelet-rich plasma was added to 500 μl of platelet wash buffer (140 mmol l^−1^ NaCl; 10 mmol l^−1^ NaHCO_3_; 2.5 mmol l^−1^ KCl; 0.5 mmol l^−1^ Na_2_HPO_4_; 1 mmol l^−1^ MgCl_2_; 6.46 mmol l^−1^ tri-sodium citrate dihydrate; 0.1% dextrose; 0.35% bovine serum albumin; pH 6.5) containing 3 U ml^−1^ Apyrase. Platelets were pelleted by centrifugation at RT at 500*g* for 15 min, and resuspended in 400 μl of sterile Tyrode's HEPES (134 mmol l^−1^ NaCl; 12 mmol l^−1^ NaHCO_3_; 2.9 mmol l^−1^ KCl; 0.34 mmol l^−1^ Na_2_HPO_4_; 1 mmol l^−1^ MgCl_2_; 1 mmol l^−1^ CaCl_2_; 10 mmol l^−1^ HEPES; 0.9% dextrose; 0.35% bovine serum albumin; pH 7.4; 1.125 mmol l^−1^ Gly-Pro-Arg-Pro). Platelets were incubated with CD62P-FITC antibody (BD Pharmingen 1:500) in the presence of SNP (36 μmol l^−1^) or not, and subsequently activated with murine α thrombin (0.01 U ml^−1^) during 15 min. Surface expression of CD62P on platelets was analysed by mean fluorescence of platelet populations between conditions using a FACScalibur (BD Biosciences) flow cytometer^57^.

### Blood clotting

Male and female mice (12 weeks old) were anaesthetized with isoflurane, 0.5 cm of the tail tip was removed with a sharp razor blade, and the tail tip was immediately put into fresh pre-warmed PBS of 37 °C. The latency until the tail stopped bleeding was visually scored and recorded with a chronometer by an observer blinded to the genotype.

### Telemetric BP measurements

BP measurements in conscious, freely moving mice were made using implantable, miniaturized mouse BP transmitters (model PhysioTel PA-C10, Data Science International)^58^. Briefly, mice weighing at least 20 g were anaesthetized with isoflurane, the left carotid artery was made accessible, and a catheter tip was inserted into the artery just before the entry of the aortic branch. The telemetric device was placed under the abdominal skin. After surgery, the mice were allowed to recover for 7 days before starting the measurements. Radiofrequency signals from the transmitters were converted to serial bit streams and data were collected and stored to disk using the Dataquest A.R.T. data acquisition system (Data Sciences International). Ambient barometric pressure was also measured and subtracted from the telemeter pressure by the Dataquest A.R.T. software to compensate for changes in atmospheric pressure^58^. Transducer sampling frequency was at 1,000 Hz and measurements were acquired in a continuous matter over 24 h. L-NAME (100 mg kg^−1^), SNP (1.5 mg kg^−1^), DETA-NO (60 mg kg^−1^), cinaciguat (300 μg kg^−1^) and the vehicle controls (PBS or for cinaciguat 20% Cremophor (Fluka)+20% diethylene-glycol-monoethyl-ether (Sigma) in PBS) were all administered intravenously (IV). BAY 41-2772 (4 mg kg^−1^) and vehicle control (20% Cremophor (Fluka)+20% propylene glycol monomethyl ether (PGME (Sigma) in PBS)) were administered IP. Murine TNF (480 μg kg^−1^) was administered IV. For 24-h BP and HR, averages of 1 h intervals were used for statistical analysis. For SNP measurements, averages of 1-min intervals of continuous data acquired at 1–15 min post injection were used for statistical analysis. For mTNF measurements, averages of 5-min intervals of continuous data acquired 1–8 h post injection were used for statistical analysis. For measurement of all other administrations, averages of 1-min intervals of continuous data acquired at 1–25 min post injection were used for statistical analysis.

### Non-invasive BP measurements

SBP and HR were measured non-invasively in awake mice by tail-cuff using a Hatteras MC4000 Blood Pressure Analysis System (model MC4000MSP, Hatteras Instruments, Inc.)^59^. Mice were habituated to the BP measurement device for 7 days. To determine basal BP, mice were measured for 15 consecutive cycles per day during 5 days. L-NAME (100 mg kg^−1^), SNP (1.5 mg kg^−1^), DETA-NO (60 mg kg^−1^), BAY 41-2772 (4 mg kg^−1^), cinaciguat (30 μg kg^−1^) and the vehicle controls (PBS or for the BAY-compounds 20% Cremophor (Fluka)+20% diethylene-glycol-monoethyl-ether (Sigma) in PBS) were all administered IP. After SNP administration, SBP measurements were recorded from 5 to 15 min post injection, for L-NAME and DETA-NO recordings were made from 1 to 25 min post injection and for BAY-compounds recordings were made from 10 to 25 min post injection.

### Aortic rings

The thoracic aorta from apo-sGC mice and WT littermates of both genders were studied in parallel. For isometric tension recording, ring segments (2 mm length) were mounted in a Mulvany–Halpern myograph organ bath containing physiological Krebs–Ringer bicarbonate solution at 37 °C gassed with 95% O_2_/5% CO_2_. Following a 30-min equilibration, the aortic rings were gradually stretched to a stable preload of 0.5 g and treated three times with 120 mmol l^−1^ K^+^ and 0.06 nmol l^−1^ phenylephrine. As soon as a stable contraction was obtained, the effects of cumulative additions of Ach (1 nmol l^−1^–10 μmol l^−1^), SNP (1 nmol l^−1^–10 μmol l^−1^), *S*-nitroso-*N*-acetylpenicillamine (1 nmol l^−1^–10 μmol l^−1^), NO gas (10 nmol l^−1^–0.1 mmol l^−1^), BAY 41-2272 (1 nmol l^−1^–1 μmol l^−1^, Alexis Biochemicals) and BAY 58-2667 (1 nmol l^−1^–1 μmol l^−1^) were recorded.

### Statistical analysis

All data are shown as mean±s.e. For pairwise analysis, Student's *t*-tests were used. Multiple-group comparisons were made using one- or two-way analysis of variance (ANOVA), as appropriate (see Supplementary Table 2 for respective experiment, number of samples and statistics used). Multiple comparison was performed after obtaining a significant ANOVA F-test. Multiple testing correction was done using the Bonferroni method. IC_50_ values were calculated using a nonlinear regression curve fitting model with variable slope. Student's *t*-tests, one- and two-way ANOVA were performed using 6.0 f for Mac (GraphPad Software, http:// www.graphpad.com).

Repeated measurements data were analysed by fitting the following linear mixed model: y_ijkl_=μ+g_i_+c_j_+t_k_+gc_ij_+gt_ik_+ct_jk_+gct_ijk_+e_ijkl_, where *y*_*ijkl*_ is the phenotypic value of the *l*th individual; *μ* is the mean term; *g*_*i*_ is the effect of the *i*th genotype (*i*=1…2;WT and KI for telemetric data and *i*=1…3; WT, HE and KI for tail-cuff data); *c*_*j*_ is the effect of the *j*th treatment (*j*=1…2; compound and control); *t*_*k*_ is the effect of the *k*th time point and *e*_*ijkl*_ is the residual effect, *e*_*ijkl*_ ∼*N*(0, σ_e_^2^) with σ_e_^2^ being the residual variance. Times of measurements were equally spaced and various ways of modelling the correlation structure (uniform, autoregressive order 1 (AR1) or 2 (AR2), and antedependence order 1 and 2) were compared in the residual maximum likelihood (REML) framework as implemented in Genstat^60^. Selection of the best model fit was based on a likelihood ratio test statistic and the Aikake Information coefficient. When residuals from the analysis indicated increasing variance over time, this was modelled directly by specifying that heterogeneity is to be introduced into model. Significance of the fixed main and interaction effects was assessed by either a Wald test or a F test.

## Additional information

**How to cite this article:** Thoonen, R. *et al*. Cardiovascular and pharmacological implications of haem-deficient NO-unresponsive soluble guanylate cyclase knock-in mice. *Nat. Commun.* 6:8482 doi: 10.1038/ncomms9482 (2015).

## Supplementary Material

Supplementary Information Supplementary Figures 1-10 and Supplementary Tables 1-9
