# Supplementary material for: Cardiovascular and pharmacological implications of haem-deficient NO-unresponsive soluble guanylate cyclase knock-in mice
Source: Nat Commun. 2015 Oct 7;6:8482. doi: 10.1038/ncomms9482 (PMC4699393; doi:10.1038/ncomms9482)
Supplement: Supplementary Information — Supplementary Figures 1-10 and Supplementary Tables 1-9 [file ncomms9482-s1.pdf]

SUPPLEMENTARY INFORMATION

Supplemental Figures and Figure Legends

Supplementary Figure 1

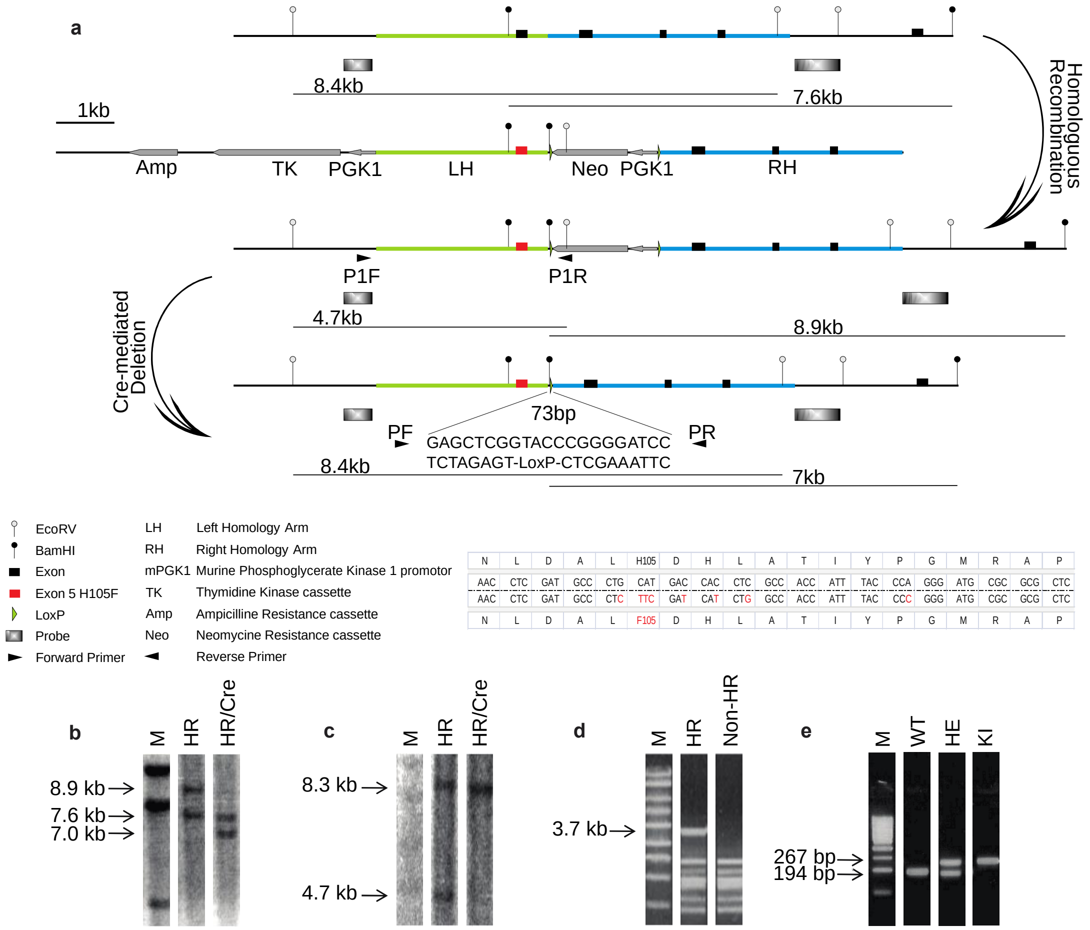

Supplementary Figure 1. Generation and genotyping of the apo-sGC (KI) mice.
(**a**) Targeting strategy for the generation of apo-sGC mice and table with mutated codons in exon 5. From top to bottom: WT sGC β1 locus; the NotI-linearized pPNTLox-sGCβ1 targeting vector; the genomic sGCβ1 allele after homologous recombination; the genomic sGCβ1 allele after Cre-mediated recombination. Restriction sites, fragment lengths, screening strategy and mutated nucleotides and amino acid sequence are as depicted. (**b**) Southern Blot analysis of genomic ES-cell DNA from the homologous recombinant (HR) clone, and the HR clone after in vitro Cre-recombination (HR/Cre), digested with BamHI and hybridized with a probe flanking the right homology arm. M: molecular weight marker (Eurogentec) (**c**) Southern Blot analysis of genomic ES-cell DNA from the HR clone, and the HR/Cre clone, digested with EcoRV and hybridized with a probe flanking the left homology arm. (**d**) PCR on recombinant ES-cell DNA with P1F and P1R generating a 3.7 kb fragment only in homologous recombinant ES-cell clones with non-tandem integrants. (**e**) PCR on genomic mouse tail DNA from Wild type (WT), heterozygous (HE) and apo-sGC (KI) mice, generating a 267 bp fragment from the mutated allele and a 194bp fragment from the WT allele.

Supplementary Figure 2

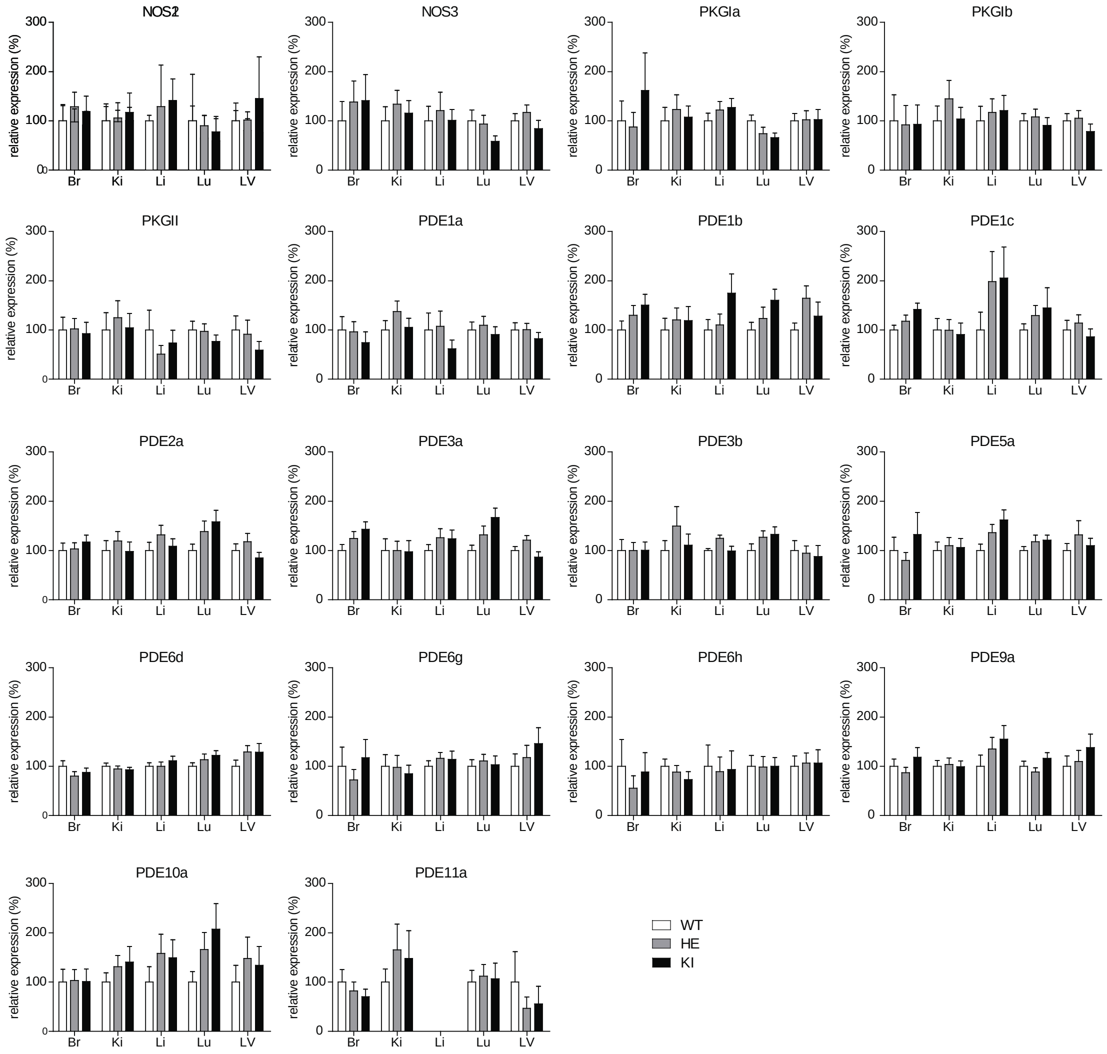

Supplementary Fig. 2. Quantitative RT-PCR of mRNA's encoding proteins in the NO-cGMP signaling pathway. nNOS: neuronal NOS, eNOS: endothelial NOS, iNOS: inducible NOS, PKG: cGMP-dependent protein kinase (isoforms Iα, Iβ, and II), PDE: phosphodiesterase (isoforms 1a, 1b, 1c, 2a, 3a, 3b, 5a, 6d, 6g, 6h, 9a, 10a, and 11a). brain (BR), liver (LI), left ventricle (LV), kidney (KI) and lung tissue (LU) . Data are presented as mean ± SE relative to WT. No significant differences were found between genders or genotypes by one-way ANOVA (n=6 for all genotypes).

Supplementary Figure 3

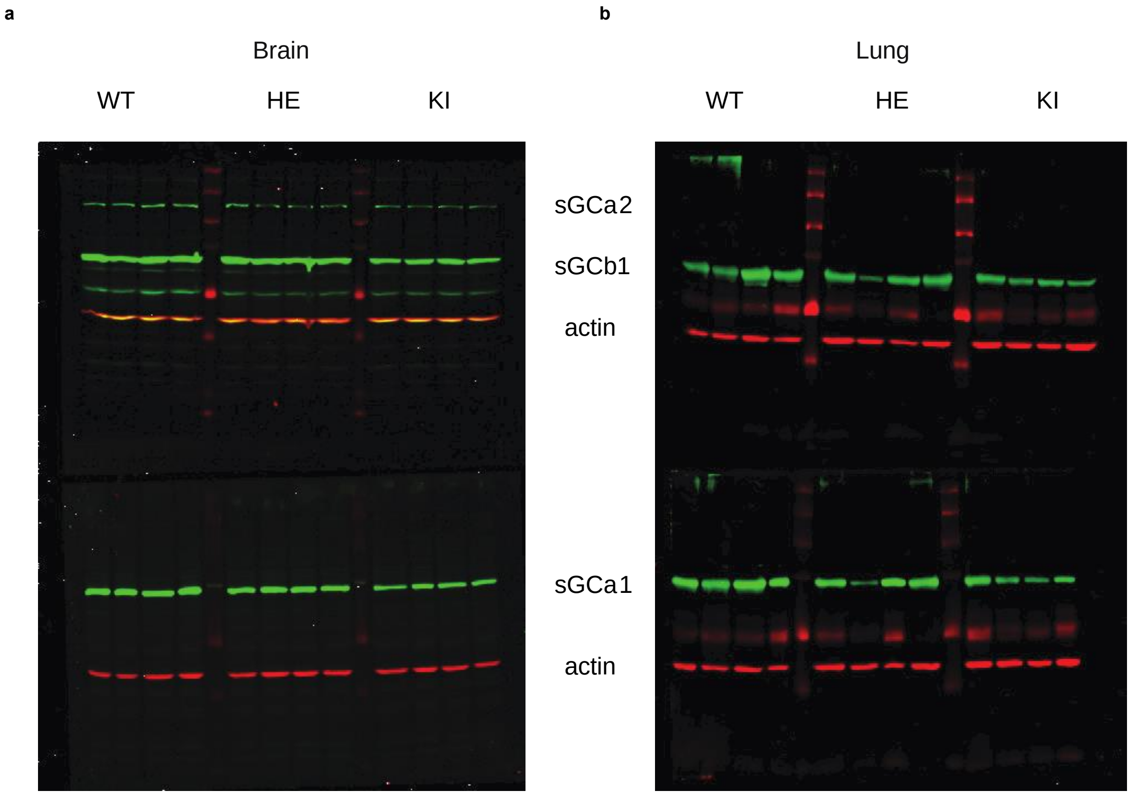

Supplementary Fig. 3. (a) Original image of representative immunoblot of sGC subunits in brain homogenates of WT, HE, and KI mice as shown in figure 1b. (b) Representative immunoblot of sGC subunits in lung homogenates of WT, HE, and KI mice. (n=4 for all genotypes, 3 experimental repeats)

Supplementary Figure 4

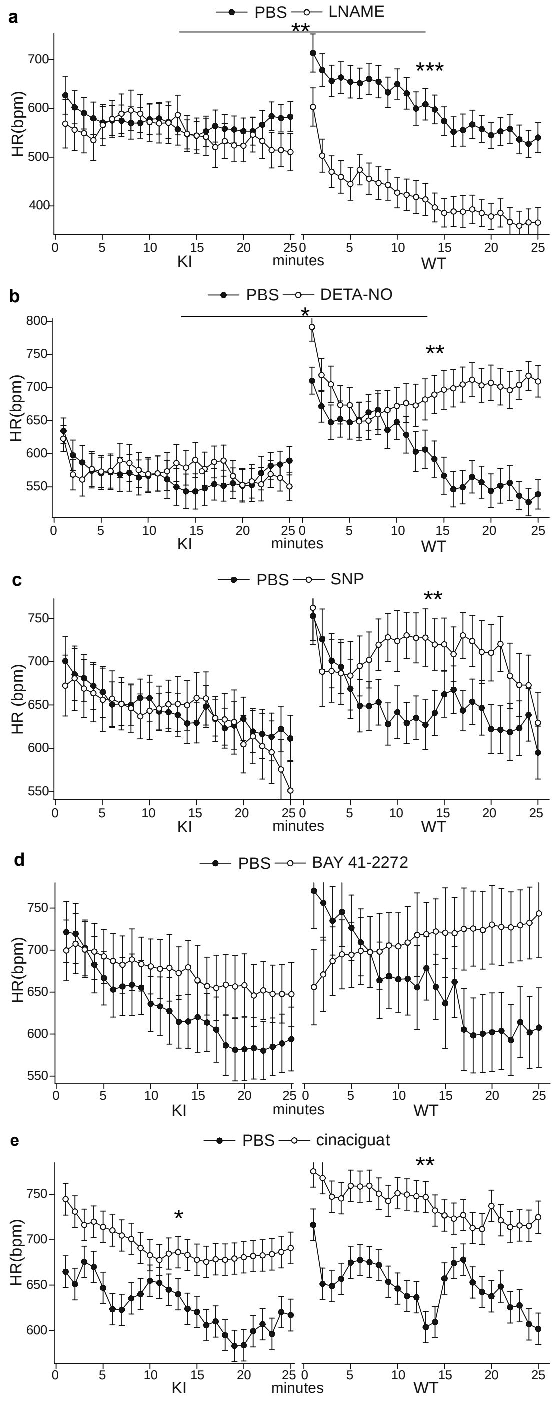

Supplementary Fig. 4. Heart rate of apo-sGC (KI) mice does not respond to endogenous and exogenous NO or BAY 41-2272, but has a stronger response to cinaciguat. Telemetric measurements of HR (**a-e**) ± s.e.m. in KI and WT mice 1-25 min after challenge with PBS IV (10 ml kg^{-1}; KI: n=9, WT: n=8) (**a-b**), PBS IP (10 ml kg^{-1}; KI: n=10, WT: n=7) (**c**), L-NAME (100 mg kg^{-1} IV; KI: n=4, WT: n=7) (**a**), DETA-NO (60 mg kg^{-1} IV; KI: n=9, WT: n=7) (**b**), SNP (1.5 mg kg^{-1} IP, KI: n=6, WT: n=5) (**c**), vehicle IP (10ml kg^{-1}; KI: n=9, WT: n=5) (**d**), vehicle IV (10ml kg^{-1}; KI: n=7, WT: n=7) (**e**), BAY 41-2772 (4mg kg^{-1} IP; KI: n=9, WT: n=5) (**d**), or cinaciguat (300mg kg^{-1} IV; KI: n=7, WT: n=8) (**e**). * $P<0.05$; ** $P<0.01$ and *** $P<0.001$ compared to WT; see Table S1-S5 with descriptive statistics for L-NAME, DETA-NO, SNP, BAY 41-2272 and cinaciguat, respectively.

Supplementary Figure 5

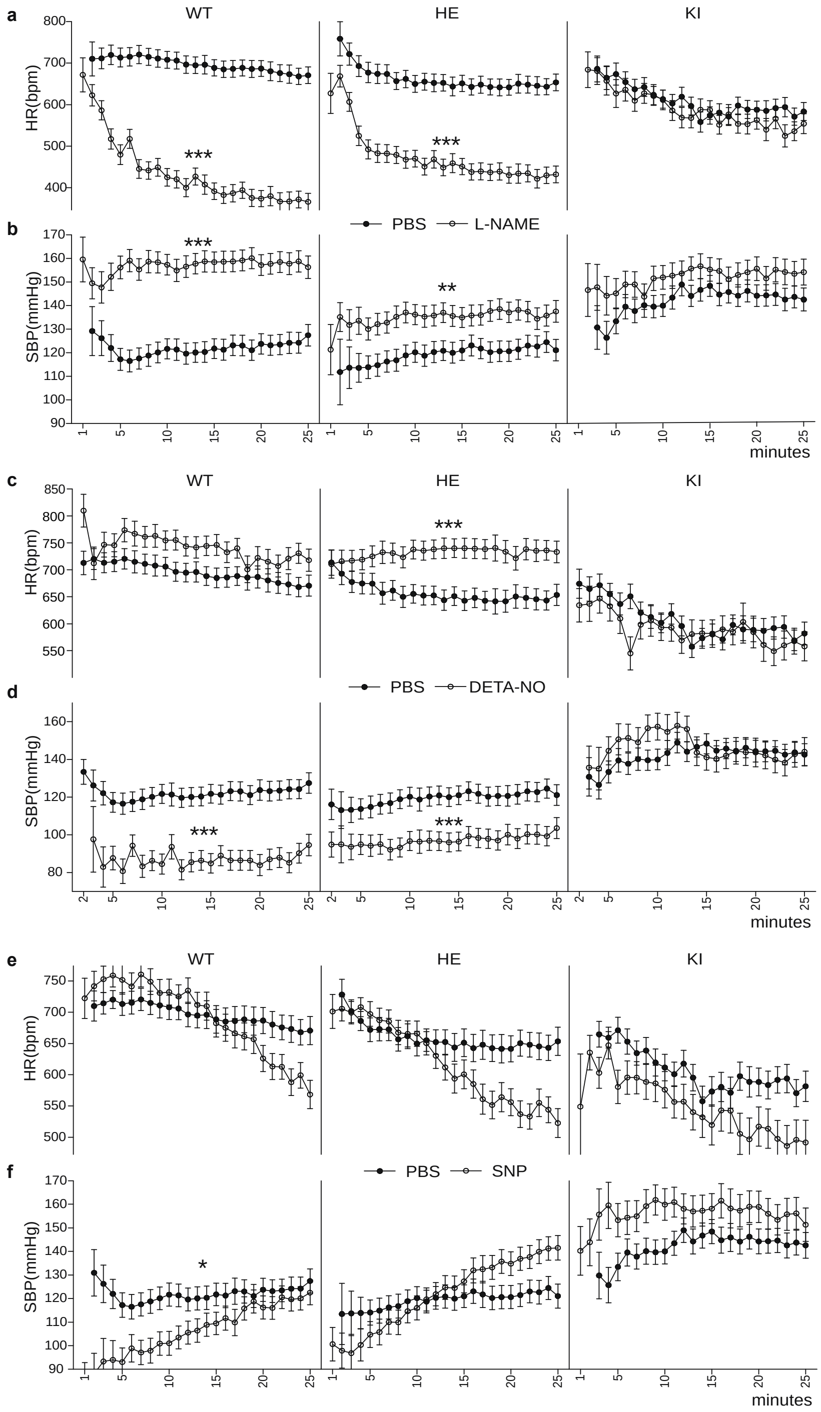

Supplementary Fig. 5. Heart rate and SBP of apo-sGC (KI) mice, obtained by tail cuff measurements do not respond to endogenous and exogenous NO. HR (**a**, **c**, e) and SBP (**b**, **d**, **f**) ± SE for WT, HE and KI mice 1-25 min after L-NAME (100 mg kg^{-1} IP; WT: n=15, HE: n=16, KI: n=11) (**a**, **b**), DETA-NO (60 mg kg^{-1} IP; WT: n=15, HE: n=16, KI: n=8) (**c**, **d**), SNP (1.5 mg kg^{-1} IP; WT: n=16, HE: n=15, KI: n=9) (**e**, **f**), and PBS IP (10 ml kg^{-1} IP; WT: n=16, HE: n=16, KI: n=14) (**a** – **f**). * P<0.05; ** P<0.01 and *** P<0.001 compared to WT; see Table S1, S2 and S3 with descriptive statistics for L-NAME, DETA-NO and SNP, respectively.

Supplementary Figure 6

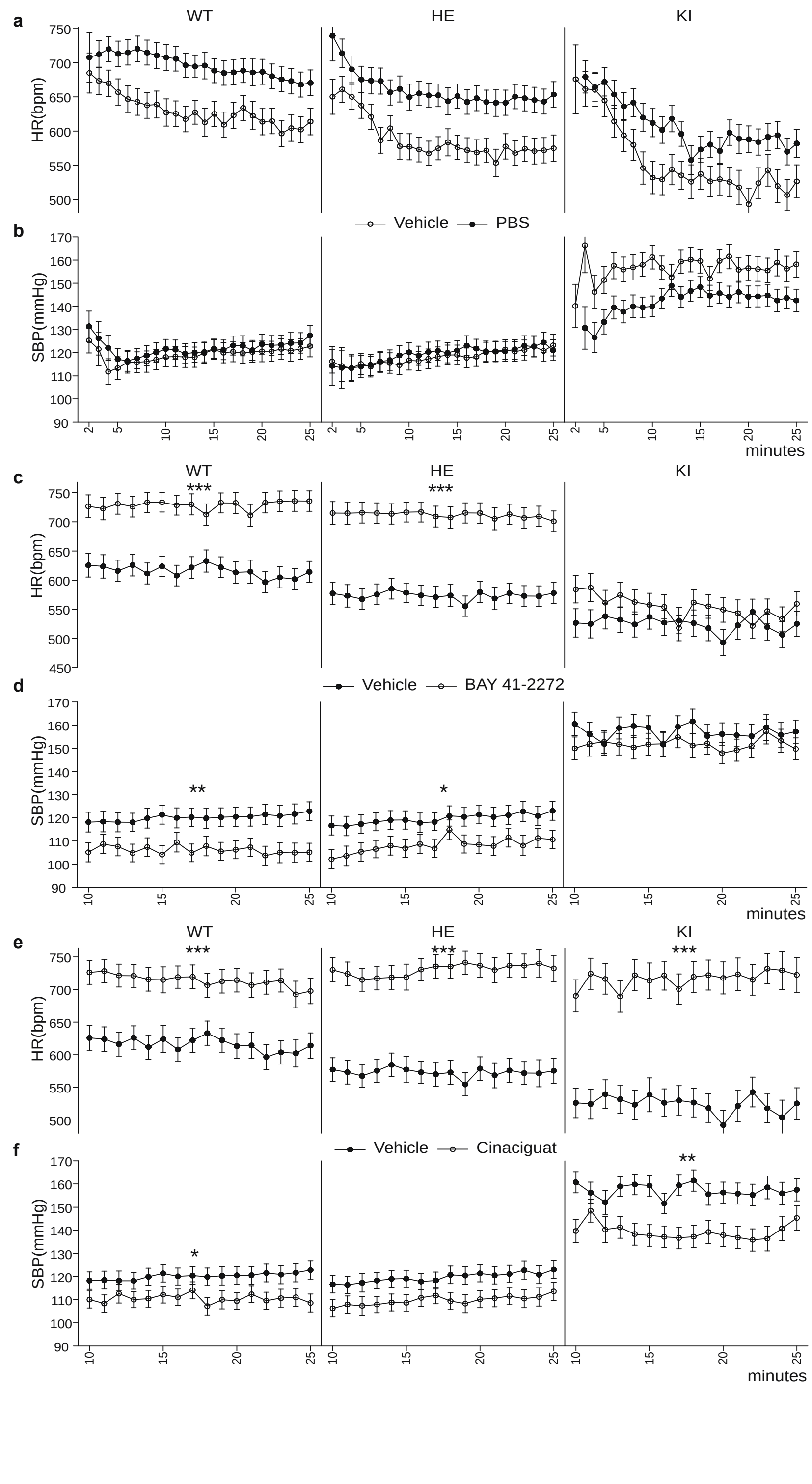

Supplementary Fig. 6. Heart rate and SBP of apo-sGC (KI) mice, obtained by tail-cuff measurements do not respond to BAY 41-1272 and have an increased response to cinaciguat (BAY58-2667). HR (**a**, **c**, **e**) and SBP (**b**, **d**, **f**) ± s.e.m. for WT, HE and KI mice 1-25 min after challenge with PBS (10 ml kg^{-1} IP; WT: n=16, HE: n=16, KI: n=14) and vehicle (10 ml kg^{-1} IP; WT: n=15, HE: n=16, KI: n=10) (**a**, **b**), and 10-25 min after challenge with BAY 41-2272 (4 mg kg^{-1} IP; WT: n=16, HE: n=16, KI: n=11) (**c**, **d**), cinaciguat (300 µg kg^{-1} IP; WT: n=16, HE: n=16, KI: n=9) (**e**, **f**), and vehicle (10 ml kg^{-1} IP; WT: n=15, HE: n=16, KI: n=10) (**c-f**). ** $P<0.01$ compared to WT; see Table S4 and S5 with descriptive statistics for BAY 41-2272 and cinaciguat, respectively.

Supplementary Figure 7

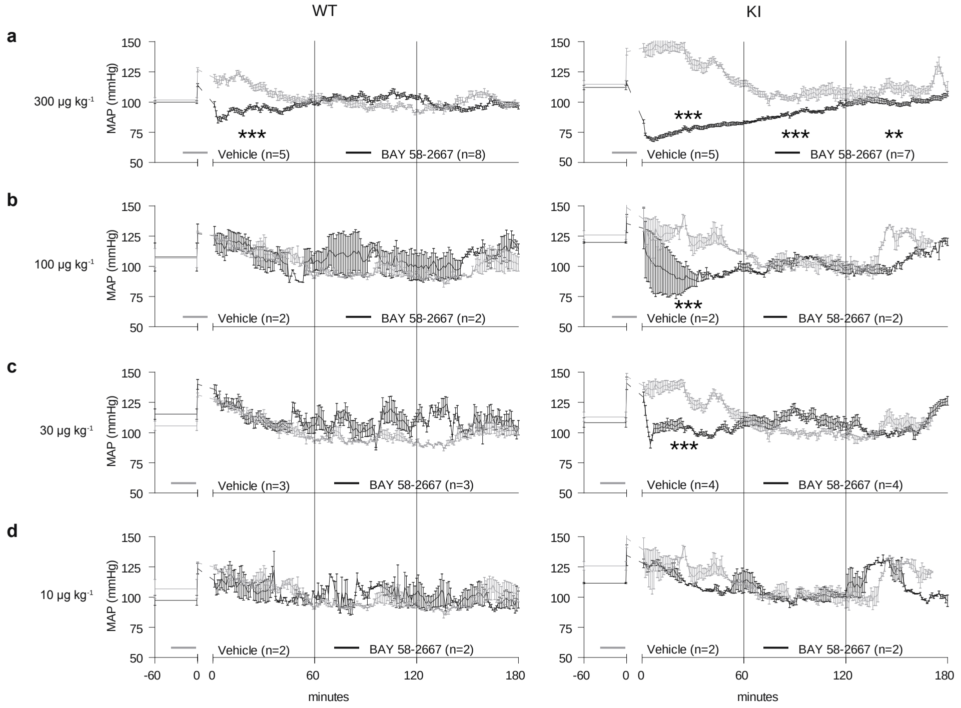

Supplementary Fig. 7. MAP dose response for cinaciguat IV at doses of 10, 30, 100 and 300 µg kg^{-1} in WT and apo-sGC mice, measured telemetrically in conscious animals up to 180 minutes after challenge **: P<0.01 and ***: P<0.001 BAY 58-2667 vs. vehicle by 2 way ANOVA (n as indicated on the figure)

Supplementary Figure 8

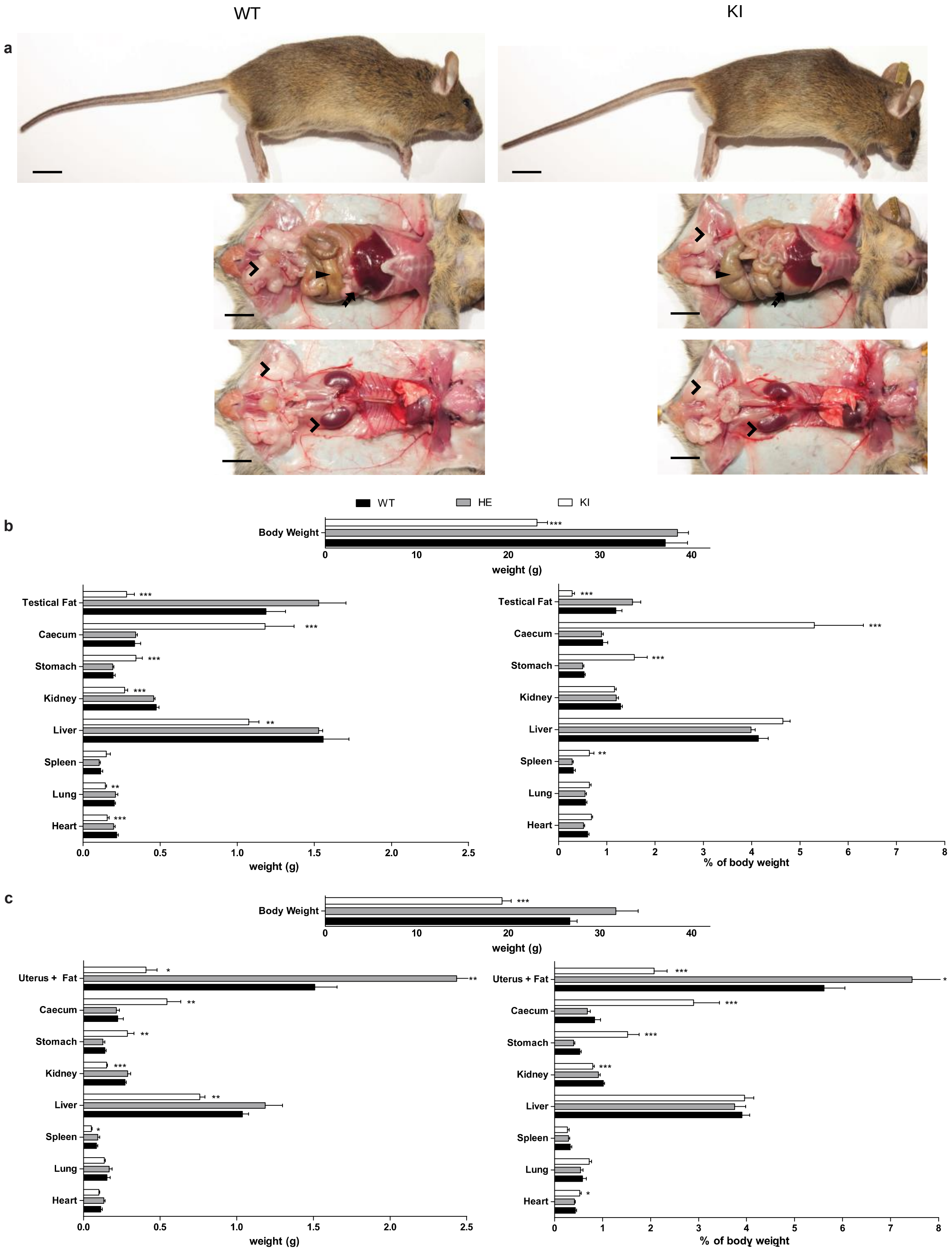

Supplementary Fig. 8. General morphological appearance of apo-sGC (KI) mice, dissection pictures and absolute and relative weights of organs. (**a**) Upper panel: pictures of 10 weeks old male WT and KI mice. Middle panel: picture showing the abdominal cavity after opening of the abdominal muscle layer of a male WT and a male KI mouse. Lower panel: dissection pictures of abdominal

cavity of male WT and KI mouse after removal of the guts and opening of the chest. Scale bar: 1 cm; open arrow: fatty tissue; arrowhead: caecum; arrow: stomach. (**b-c**) Body weight, absolute organ weights and relative organ weights of (**b**) male and (**c**) female WT, HE and KI mice (n=7 for each genotype). * *P*<0.05, ** *P*<0.01, and *** *P*<0.001; one-way ANOVA followed by Bonferroni post-tests compared to WT.

Supplementary Figure 9

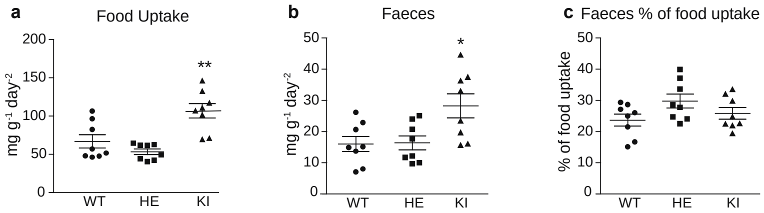

Supplementary Fig. 9. Scatterplot with mean daily metabolic recordings measured in male apo-sGC (KI) mice and littermate controls expressed per g of body weight per day ± s.e.m. Apo-sGC and HE mice have similar serum glucose (**a**) and triglyceride (**b**) levels as WT mice but have reduced fructosamine (**c**) levels. Apo-sGC mice display significantly increased daily food uptake (**d**), and faeces excretion (**e**). Faeces excretion relative to food uptake is similar for all genotypes (**f**). * *P*<0.05, and ** *P*<0.01; one-way ANOVA followed by Dunnett's multiple comparison test. (serum glucose and triglycerides: WT: n=8, HE: n=8 and KI: n=7, respectively; fructosamine: WT: n=14, HE: n=13 and KI: n=14, respectively).

Supplementary Figure 10

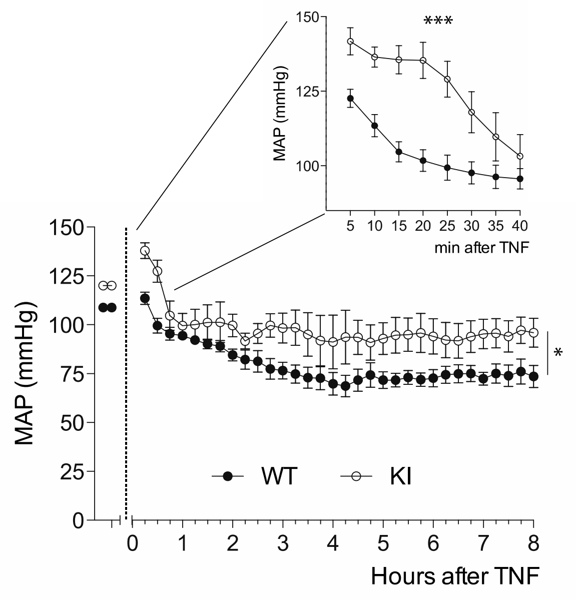

Supplementary Fig. 10. Absolute MAP ± SE measured telemetrically in KI (○) and WT mice (●) 0-8h after challenge with an LD100 of murine TNF IV (480ug kg^{-1}). The insert in represents the response in MAP during the first 30 minutes after TNF challenge. *** P<0.001: changes in MAP after challenge compared to control

Supplementary Tables

Supplementary Table 1.

Sequence 5'-3'	Target	Sequence 5'-3'	Target
CCCCTGGTCAGGTTCCTAAG	sGCa1 F	ATATGCAGGGAGTCGTGTACG	PDE10a F
GGAGACTCCCTTCTGCATTCT	sGCa1 R	GTCGGCTTTTGTGGCTATCC	PDE10a R
TGCATCTCTCAGACATCCCTATTC	sGCa2 F	TGGCGTTGTGGACGATGAG	PDE2a F
TGGGCCTTGGCTTGCTC	sGCa2 R	CGCGATAGAAAAGCGGATGG	PDE2a R
TGCTGGTGATCCGCAATTATG	sGCb1 F	TCCCAGTCAGGAACCAGCAT	PDE3a F
GGTTGAGGACTTTGCTTGCAG	sGCb1 R	CAAGTTGCTTACGGCCCTC	PDE3a R
ACCCTGCGGGATTTACAGTAT	PKGI(b) F	CAGTAGCTTGATGGGTGCTTT	PDE3b F
CGTCCTTCTGATCCAACTCCA	PKGI(b) R	CCCTTGTGAAGTTTTCGATCTCC	PDE3b R
ATCCGAGAGGTCGAAGGATCT	PKGI(a) F	CGGCCTACCTGGCATTCTG	PDE5 F
ATTCCACGGGGTACATACAGT	PKGI(a) R	GCAAGGTCAAGTAACACCTGATT	PDE5 R
AACAGGGCAGCTACGTCATTA	PKGII F	GGCAGCTAGGAACAGCAGG	PDE9a F
TTGGGATGGATGATAGCAACTTC	PKGII R	GGTACTTGGGGTAAGTGGGGA	PDE9a R
CTGGTGAAGGAACGGGTCAG	NOS-1 F	TTTCAAAGAGAGTGTGGAGAAATCG	PDE11a F
CCGATCATTGACGGCGAGAAT	NOS-1 R	GCATCGCTGACATTCACAGGAA	PDE11a R
GTTCTCAGCCCAACAATACAAGA	NOS-2 F	CCTGCTGCTCTCAAGGTTGTT	RPL13a F
GTGGACGGGTCGATGTCAC	NOS-2 R	TGGTTGTCACTGCCTGGTACTT	RPL13a R
CACCTACGACACCCTCAGTG	NOS-3 F	GAAACTCTGCTTCGCTGCATT	HMBS F
CTTGACCCAATAGCTGCTCAG	NOS-3 R	TGCCCATCTTTCATCACTGTATG	HMBS R
CCAGACTGACTCCGTCCCAT	PDE1a F	TCTACCGTGAATCTTGGCTGTAAA	TBP F
CCATTTTGCGTGTGAAAGTTGA	PDE1a R	TTCTCATGATGACTGCAGCAAA	TBP R
AGTTCCGAAGCATCGTGCAT	PDE1b F	ATGCACGCAGAAAGAAATAGCAA'	B2M F
AGGTTCTTGAGACAGTTGTGGA	PDE1b R	AGCTATCTAGGATATTTCCAATTTTTGAA	B2M R
ACGTCCCAGAGGTTACGGT	PDE1c F	GCTTCTAGGCGGACTGTTACTGA	ACTB F
GGCTGCATATTCCAGATTCTTCT	PDE1c R	GCCATGCCAATGTTGTCTCTTAT	ACTB R

Supplementary Table 1. QPCR primers for respective gene detection.

Supplementary Table 2.

Figure	Experiment type	N	Statistical Test
**1 A**	sGC mRNA	WT: n=6; HE: n=6; KI: n=6	one way - ANOVA
**1 B**	sGC immunoblot	WT: n=4; HE: n=4; KI: n= 4	one way - ANOVA
**1 C**	Basal	WT: n=12, HE: n=11, KI: n=11	
	DETA-NO	WT: n=7, HE: n=7, KI: n=7	one way - ANOVA
**1 D-E**	sGC activity baseline	WT: n=20, KI: n=17	
	DETA-NO	WT: n=20, KI: n=17	
	Cinaciguat	WT: n=18, KI: n=15	one way - ANOVA
**2 A**	CHO basal activity	N=6 experimental repeats of 4 biological repeats/experiment	Students t-test
**2 B-E**	CHO stimulated activity	N=6 experimental repeats of 4 biological repeats/experiment	Students t-test
**3 A**	Tail bleeding time	WT: n=29, HE: n=30 and KI: n=27	one way - ANOVA
**3 B**	Platelet activation	WT: n=4, KI: n=4	two way - ANOVA
**3 C**	Tail-cuff: SBP male	WT: n=42, HE: n=25 and KI: n=39	
	Tail-cuff: SBP female	WT: n=38, HE: n=24 and KI: n=36	one way - ANOVA
**3 D-E**	Telemetry: SBP and HR	WT: n=8, KI: n=7	Students t-test
**4 A-B**	Telemetry: MAP: PBS IV	WT: n=8, KI: n=9	REML (table 3-4)
**4 C**	Telemetry: MAP: PBS IP	WT: n=7, KI: n=10	REML (table 5)
**4 A**	Telemetry: MAP: L-NAME IV	WT: n=7, KI: n=4	REML (table 3)
**4 B**	Telemetry: MAP:DETA-NO IV	WT: n=7, KI: n=9	REML (table 4)
**4 C**	Telemetry: MAP: SNP IP	WT: n=5, KI: n=6	REML (table 5)
**4 D**	Telemetry: MAP: vehicle IP	WT: n=5, KI: n=9	REML (table 6)
**4 E**	Telemetry: MAP: vehicle IV	WT: n=7, KI: n=7	REML (table 7)
**4 D**	Telemetry: MAP: BAY 41-2272	WT: n=5, KI: n=9	REML (table 6)
**4 E**	Telemetry: MAP: Cinaciguat	WT: n=8, KI: n=7	REML (table 7)
**5 A**	Relaxation after ACh	WT: n=6, KI: n=6	
**5 B**	Relaxation after SNP	WT: n=6, KI: n=6	
**5 C**	Relaxation after SNAP	WT: n=6, KI: n=6	
**5 D**	Relaxation after NO-gas	WT: n=6, KI: n=6	
**5 E**	Relaxation after BAY 41-2272	WT: n=6, KI: n=7	
**5 F**	Relaxation after cinaciguat	WT: n=6, KI: n=7	Students t-test
**6 A**	Kaplan-Meier survival plots male	WT: n=633, HE: n=1370, KI: n=545	Mantel-Cox test
**6 B**	Growth curve male	WT: n= 56, HE: n= 129, KI: n =69	REML AR2 analysis followed by F-test
	Growth curve female	WT: n= 63, HE: n= 121, KI: n= 52	
**6 C**	Serum glucose	WT: n=8, HE: n=8, KI: n=7	
**6 D**	Plasma triglycerides	WT: n=8, HE: n=8, KI: n=7	
**6 E**	Fructosamine	WT: n=14, HE: n=13, KI: n=14	one-way ANOVA
**7 A-B**	Telemetry MAP and HR after TNF	WT: n=8, KI: n=7	REML (table 8-9)
**7 C-D**	Survival after TNF		Mantel-Cox

Supplementary Table 2. Table with statistical overview for all experiments performed

Supplementary Table 3.

TELEMETRY	MAP					HR				
Fixed term	Wald statistic	n.d.f.	F statistic	d.d.f.	F pr	Wald statistic	n.d.f.	F statistic	d.d.f.	F pr
**Time**	46.61	24	1.74	137.2	0.025	107.34	24	4.05	147.3	<0.001
**Genotype**	0.19	1	0.19	20	0.668	5.51	1	5.51	20.4	0.029
**Treatment**	19.46	1	19.46	20	<0.001	13.7	1	13.7	20.4	0.001
**Time.Genotype**	19.41	24	0.73	137.2	0.819	36.17	24	1.37	147.3	0.134
**Time.Treatment**	18.28	24	0.68	137.2	0.861	26.36	24	0.99	147.3	0.477
**Genotype.Treatment**	7.5	1	7.5	20	0.013	4.92	1	4.92	20.4	0.038
**Time.Genotype.Treatment**	12.56	24	0.47	137.2	0.983	28.64	24	1.08	147.3	0.372

Statistical analysis of HR and MAP obtained via telemetry, 1-25 min after injection of L-NAME IV (WT: n=7; KI: n=4) compared to PBS IV (WT: n=8; KI: n=9). The repeated measurements data were analyzed using a linear mixed model restricted maximum likelihood (REML). Covariance over time has been modeled as AR1 (autoregressive order 1) and variance heterogeneity across time points was accounted for. The significance of genotype effects on the changes in MAP and HR caused by the treatment were assessed by an F test.

TAIL-CUFF		SBP					HR				
	Fixed term	Wald statistic	n.d.f.	F statistic	d.d.f.	F pr	Wald statistic	n.d.f.	F statistic	d.d.f.	F pr
	**Time**	36.69	23	1.55	497.3	0.05	476.16	23	20.08	480.1	<0.001
	**Genotype**	17.2	2	8.6	81.7	<0.001	4.28	2	2.14	82.9	0.124
	**Treatment**	33.73	1	33.73	81.6	<0.001	118.2	1	118.2	82.8	<0.001
**A**	**Time.Genotype**	44.91	46	0.95	717	0.574	51.58	46	1.09	693.3	0.326
	**Time.Treatment**	20.8	23	0.88	503	0.628	90.08	23	3.8	482.5	<0.001
	**Genotype.Treatment**	9.12	2	4.56	81.7	0.013	42.05	2	21.03	83	<0.001
	**Time.Genotype.Treatment**	52.92	45	1.14	746.3	0.245	64.45	45	1.39	731.9	0.049
	**Genotype**	17.44	2	8.72	81.7	<0.001	3.4	2	1.7	82.9	0.189
	**Treatment**	33.47	1	33.47	81.8	<0.001	112.54	1	112.54	83.1	<0.001
	**GT1**	5.64	1	5.64	81.4	0.02	4.63	1	4.63	82.1	0.034
	**GT2**	3.64	1	3.64	82	0.06	35.56	1	35.56	83.9	<0.001
**B**	**Time**	36.6	23	1.55	497.4	0.051	483.38	23	20.38	480.1	<0.001
	**Time.Genotype**	44.91	46	0.95	729.5	0.573	51.94	46	1.09	702.6	0.313
	**Time.Treatment**	20.75	23	0.88	503.6	0.631	90.9	23	3.83	482.6	<0.001
	**Time.GT1**	23.84	23	1.01	498.4	0.454	26.91	23	1.13	479	0.303
	**Time.GT2**	29.08	22	1.29	533.5	0.172	37.54	22	1.66	524	0.03

Statistical analysis of SBP and HR obtained via tail-cuff, 2-25 min after injection of L-NAME IP (WT: n=15; HE: n=16; KI: n=11) compared to PBS IP (WT: n=16; HE: n=16; KI: n=14). The repeated measurement data were analyzed using a linear mixed model REML. Covariance over time has been modeled as AR2 (autoregressive order 2) and variance heterogeneity across time points was accounted for. Fixed effects (A), and fixed effects including the linear contrasts GT1 (comparing WT and HE means at the two levels of treatment) and GT2 (comparing WT and KI means at the two levels of treatment) (B) were assessed by F tests.

Supplementary Table 4.

TELEMETRY	MAP					HR				
**Fixed term**	**Wald statistic**	**n.d.f.**	**F statistic**	**d.d.f.**	**F pr**	**Wald statistic**	**n.d.f.**	**F statistic**	**d.d.f.**	**F pr**
**Time**	64.24	24	2.47	189.8	<0.001	70.4	24	2.73	210.3	<0.001
**Genotype**	51.7	1	51.7	28.3	<0.001	4.06	1	4.06	28.7	0.053
**Treatment**	21.49	1	21.49	28.3	<0.001	2.09	1	2.09	28.7	0.159
**Time.Genotype**	22.73	24	0.87	189.8	0.639	21.05	24	0.82	210.3	0.714
**Time.Treatment**	25.11	24	0.96	189.8	0.516	44.41	24	1.72	210.3	0.023
**Genotype.Treatment**	37.82	1	37.82	28.3	<0.001	6.53	1	6.53	28.7	0.016
**Time.Genotype.Treatment**	31.51	24	1.21	189.8	0.238	51.97	24	2.02	210.3	0.005

Statistical analysis of HR and MAP obtained via telemetry, 1-25 min after injection of DETA-NO IV (WT: n=7; KI: n=9) compared to PBS IV (WT: n=8; KI: n=9). The repeated measurements data were analyzed using a linear mixed model restricted maximum likelihood (REML). Covariance over time has been modeled as AR1 (autoregressive order 1) and variance heterogeneity across time points was accounted for. The significance of genotype effects on the changes in MAP and HR caused by the treatment were assessed by an F test.

TAIL-CUFF		SBP					HR				
	**Fixed term**	**Wald statistic**	**n.d.f.**	**F statistic**	**d.d.f.**	**F pr**	**Wald statistic**	**n.d.f.**	**F statistic**	**d.d.f.**	**F pr**
**A**	**Time**	33.4	22	1.47	422.6	0.08	59.92	22	2.64	432.4	<0.001
	**Genotype**	56.73	2	28.36	77.7	<0.001	39.49	2	19.75	77.3	<0.001
	**Treatment**	22.77	1	22.77	77.7	<0.001	9.26	1	9.26	77.4	0.003
	**Time.Genotype**	72.55	44	1.59	609.9	0.01	70.74	44	1.55	626.3	0.014
	**Time.Treatment**	36.82	22	1.62	427.3	0.039	31.29	22	1.38	439.1	0.12
	**Genotype.Treatment**	11.84	2	5.92	77.8	0.004	5.86	2	2.93	77.6	0.059
	**Time.Genotype.Treatment**	73.04	44	1.6	616.1	0.009	53.13	44	1.17	634.9	0.217
**B**	**Genotype**	56.8	2	28.4	77.8	<0.001	39.59	2	19.8	77.5	<0.001
	**Treatment**	22.55	1	22.55	77.9	<0.001	8.97	1	8.97	77.7	0.004
	**GT1**	0.99	1	0.99	77.6	0.322	0.5	1	0.5	77.2	0.48
	**GT2**	10.52	1	10.52	78	0.002	5.17	1	5.17	78.1	0.026
	**Time**	33.76	22	1.48	422.6	0.074	60.19	22	2.65	432.4	<0.001
	**Time.Genotype**	72.74	44	1.6	616.1	0.01	70.89	44	1.56	634.9	0.014
	**Time.Treatment**	36.75	22	1.62	431.2	0.039	31.24	22	1.38	443.3	0.12
	**Time.GT1**	20.55	22	0.9	426.1	0.59	19.98	22	0.88	438.2	0.623
	**Time.GT2**	52.49	22	2.31	433.9	<0.001	33.15	22	1.46	446.2	0.082

Statistical analysis of SBP and HR obtained via tail-cuff, 2-25 min after injection of DETA-NO IP (WT: n=15; HE: n=16; KI: n=8) compared to PBS IP (WT: n=16; HE: n=16; KI: n=14). The repeated measurement data were analyzed using a linear mixed model REML. Covariance over time has been modeled as AR2 (autoregressive order 2) and variance heterogeneity across time points was accounted for. Fixed effects (A), and fixed effects including the linear contrasts GT1 (comparing WT and HE means at the two levels of treatment) and GT2 (comparing WT and KI means at the two levels of treatment) (B) were assessed by F tests

Supplemenatary Table 5.

TELEMETRY	MAP	HR

Fixed term	Wald statistic	d.f.	Wald/d.f.	chi pr	.	Wald statistic	n.d.f.	F statistic	d.d.f.	F pr
**Time**	48.61	14	3.47	<0.001	.	36.06	14	2.36	99.2	0.007
**Genotype**	47.32	1	47.32	<0.001	.	1.92	1	1.92	22.5	0.18
**Treatment**	7.37	1	7.37	0.007	.	4.34	1	4.34	22.8	0.049
**Time.Genotype**	53.81	14	3.84	<0.001	.	15.73	14	1.03	99.1	0.43
**Time.Treatment**	36.83	14	2.63	<0.001	.	12.17	14	0.8	99.6	0.67
**Genotype.Treatment**	4.67	1	4.67	0.031	.	4.29	1	4.29	22.7	0.05
**Time.Genotype.Treatment**	43.72	14	3.12	<0.001	.	26.04	14	1.71	99.4	0.066

Statistical analysis of HR and MAP obtained via telemetry, 1-15 min after injection of SNP IP (WT: n=5; KI: n=6), compared to PBS IP (WT: n=7; KI: n=10). The repeated measurements data were analyzed using a linear mixed model restricted maximum likelihood (REML). Covariance over time has been modeled as AR1 (autoregressive order 1) and variance heterogeneity across time points was accounted for. The significance of genotype effects on the changes in MAP and HR caused by the treatment were assessed by either a Wald test (MAP) or F test (HR).

TAIL-CUFF		SBP					HR				
	**Fixed term**	**Wald statistic**	**n.d.f.**	**F statistic**	**d.d.f.**	**F pr**	**Wald statistic**	**n.d.f.**	**F statistic**	**d.d.f.**	**F pr**
	**Time**	104.24	13	7.68	186.6	<0.001	94.95	13	7.03	209.5	<0.001
	**Genotype**	43.98	2	21.99	77.1	<0.001	32.71	2	16.35	73.6	<0.001
	**Treatment**	0.22	1	0.22	77.2	0.643	2.03	1	2.03	75.5	0.158
**A**	**Time.Genotype**	30.03	26	1.1	260.9	0.337	43.35	26	1.6	296.5	0.035
	**Time.Treatment**	19.69	13	1.45	189.2	0.139	26.33	13	1.95	215.5	0.026
	**Genotype.Treatment**	8.91	2	4.45	77.5	0.015	2.13	2	1.07	77.1	0.349
	**Time.Genotype.Treatment**	47.5	25	1.82	293.8	0.011	30.4	25	1.17	324.1	0.263
	**Genotype**	43.09	2	21.55	77.3	<0.001					
	**Treatment**	0.16	1	0.16	77.7	0.69					
	**GT1**	2.35	1	2.35	76.8	0.129					
	**GT2**	5.5	1	5.5	78.3	0.022					
**B**	**Time**	106.14	13	7.82	186.6	<0.001					
	**Time.Genotype**	29.83	26	1.1	276.5	0.341					
	**Time.Treatment**	19.99	13	1.47	189.3	0.13					
	**Time.GT1**	5.92	13	0.44	187.6	0.955					
	**Time.GT2**	41.58	12	3.35	224.8	<0.001					

Statistical analysis of SBP and HR obtained via tail-cuff, 2-15 min after injection of SNP IP (WT: n=16; HE: n=15; KI: n=9) compared to PBS IP (WT: n=16; HE: n=16; KI: n=14). The repeated measurement data were analyzed using a linear mixed model REML. Covariance over time has been modeled as AR2 (autoregressive order 2) and variance heterogeneity across time points was accounted for. Fixed effects (A), and fixed effects including the linear contrasts GT1 (comparing WT and HE means at the two levels of treatment) and GT2 (comparing WT and KI means at the two levels of treatment) (B) were assessed by F tests.

Supplementary Table 6.

TELEMETRY	MAP					HR				
**Fixed term**	**Wald statistic**	**n.d.f.**	**F statistic**	**d.d.f.**	**F pr**	**Wald statistic**	**n.d.f.**	**F statistic**	**d.d.f.**	**F pr**

**Time**	65.67	24	2.52	175.5	<0.001	42.83	24	1.62	154	0.044
**Genotype**	39.5	1	39.5	23.8	<0.001	4.27	1	4.27	22.1	0.051
**Treatment**	5.13	1	5.13	23.8	0.033	2.02	1	2.02	22.1	0.169
**Time.Genotype**	34.93	24	1.34	176.6	0.144	30.93	24	1.17	155	0.279
**Time.Treatment**	32.87	24	1.26	176	0.197	49.09	24	1.85	154.4	0.014
**Genotype.Treatment**	4.55	1	4.55	23.9	0.043	0.63	1	0.63	22.1	0.437
**Time.Genotype.Treatment**	16.99	24	0.65	177.6	0.891	21.15	24	0.8	155.9	0.734

Statistical analysis of HR and MAP obtained via telemetry, 1-25 min after injection of BAY 41-2272 IP (WT: n=5; KI n=9) compared to vehicle IP (WT: n=5; KI: n=9). The repeated measurements data were analyzed using a linear mixed model restricted maximum likelihood (REML). Covariance over time has been modeled as AR1 (autoregressive order 1) and variance heterogeneity across time points was accounted for. The significance of genotype effects on the changes in MAP and HR caused by the treatment were assessed by an F test.

TAIL-CUFF		SBP					HR				
	**Fixed term**	**Wald statistic**	**n.d.f.**	**F statistic**	**d.d.f.**	**F pr**	**Wald statistic**	**n.d.f.**	**F statistic**	**d.d.f.**	**F pr**
**A**	**Time**	17.95	15	1.16	341.6	0.299	17.98	15	1.17	350.7	0.296
	**Genotype**	110.51	2	55.25	78	<0.001	49.92	2	24.96	76.9	<0.001
	**Treatment**	12.25	1	12.25	77.7	<0.001	48.38	1	48.38	76.7	<0.001
	**Time.Genotype**	45.17	30	1.46	490.4	0.057	34.56	30	1.12	500.1	0.305
	**Time.Treatment**	17.83	15	1.16	341.7	0.305	14.34	15	0.93	350.8	0.531
	**Genotype.Treatment**	0.93	2	0.47	78	0.629	8.6	2	4.3	77	0.017
	**Time.Genotype.Treatment**	26.54	30	0.86	490.4	0.685	59.61	30	1.93	500.2	0.003
**B**	**Genotype**						50.13	2	25.07	76.8	<0.001
	**Treatment**						48.89	1	48.89	77.1	<0.001
	**GT1**						0.51	1	0.51	75.9	0.476
	**GT2**						8.44	1	8.44	77.9	0.005
	**Time**						15.58	15	1.01	334.5	0.445
	**Time.Genotype**						33.41	30	1.08	482.5	0.356
	**Time.Treatment**						15.04	15	0.97	337.7	0.482
	**Time.GT1**						17.07	15	1.11	332.2	0.35
	**Time.GT2**						40.76	15	2.64	341.3	<0.001

Statistical analysis of SBP and HR obtained via tail-cuff, 10-25 min after injection of BAY 41-2272 IP (WT: n=16; HE: n=16; KI: n11) compared to vehicle IP (WT: n=15; HE: n=16; KI: n=10). The repeated measurement data were analyzed using a linear mixed model REML. Covariance over time has been modeled as AR2 (autoregressive order 2) (SBP) or AR1 (HR) and variance heterogeneity across time points was accounted for. Fixed effects (A), and fixed effects including the linear contrasts GT1 (comparing WT and HE means at the two levels of treatment) and GT2 (comparing WT and KI means at the two levels of treatment) (B) were assessed by F tests.

Supplementary Table 7.

TELEMETRY	MAP					HR				
**Fixed term**	**Wald statistic**	**n.d.f.**	**F statistic**	**d.d.f.**	**F pr**	**Wald statistic**	**n.d.f.**	**F statistic**	**d.d.f.**	**F pr**
**Time**	371.52	24	14.2	173.1	<0.001	141.27	24	5.38	166.9	<0.001
**Genotype**	0.25	1	0.25	24.6	0.619	2.54	1	2.54	23.7	0.124

**Treatment**	110.92	1	110.92	24.6	<0.001	16.83	1	16.83	23.7	<0.001
**Time.Genotype**	78.35	24	2.99	173.1	<0.001	74.75	24	2.85	166.9	<0.001
**Time.Treatment**	130.78	24	5	173.1	<0.001	52.09	24	1.99	166.9	0.007
**Genotype.Treatment**	23.63	1	23.63	24.6	<0.001	0.5	1	0.5	23.7	0.487
**Time.Genotype.Treatment**	51.26	24	1.96	173.1	0.007	65.32	24	2.49	166.9	<0.001

Statistical analysis of HR and MAP obtained via telemetry, 1-25 min after injection of cinaciguat IV (WT: n=8; KI n=7) compared to vehicle IV (WT: n=7; KI: n=7). The repeated measurements data were analyzed using a linear mixed model restricted maximum likelihood (REML). Covariance over time has been modeled as AR1 (autoregressive order 1) and variance heterogeneity across time points was accounted for. The significance of genotype effects on the changes in MAP and HR caused by the treatment were assessed by an F test.

TAIL-CUFF	MAP					HR				
**Fixed term**	**Wald statistic**	**n.d.f.**	**F statistic**	**d.d.f.**	**F pr**	**Wald statistic**	**n.d.f.**	**F statistic**	**d.d.f.**	**F pr**
**Time**	17.65	15	1.14	322.2	0.318	5.62	15	0.36	319.2	0.987
**Genotype**	83.21	2	41.61	75.8	<0.001	6.32	2	3.16	74.8	0.048
**Treatment**	13.78	1	13.78	75.6	<0.001	87.13	1	87.13	74.6	<0.001
**Time.Genotype**	51.53	30	1.66	461.9	0.016	37.68	30	1.22	458.3	0.203
**Time.Treatment**	22.47	15	1.45	322.7	0.121	20.16	15	1.3	319.6	0.198
**Genotype.Treatment**	1.29	2	0.64	75.8	0.528	5.24	2	2.62	74.9	0.08
**Time.Genotype.Treatment**	37.92	30	1.22	462.7	0.195	37.35	30	1.21	459	0.212

Statistical analysis of SBP and HR obtained via tail-cuff, 10-25 min after injection of cinaciguat IV (WT: n=16; HE: n=16; KI: n=9) compared to vehicle IV (WT: n=15; HE: n=16; KI: n=10). The repeated measurement data were analyzed using a linear mixed model REML. Covariance over time has been modeled as AR2 (autoregressive order 2) and variance heterogeneity across time points was accounted for. Fixed effects (A), and fixed effects including the linear contrasts GT1 (comparing WT and HE means at the two levels of treatment) and GT2 (comparing WT and KI means at the two levels of treatment) (B) were assessed by F tests.

Supplementary Table 8.

TELEMETRY	MAP				HR				
**Fixed term**	**Wald statistic**	**d.f.**	**Wald/d.f.**	**Chi pr**	**Wald statistic**	**n.d.f.**	**F statistic**	**d.d.f.**	**F pr**
**Time**	270.73	95	2.85	<0.001	314.74	95	3.31	1119.8	<0.001
**Genotype**	18.38	1	18.38	0.078	2.61	1	2.61	21	0.121
**Time.Genotype**	145.80	95	1.53	0.062	96.21	95	1.01	1119.8	0.451

Statistical analysis of HR and MAP obtained via telemetry, 1-8 h after injection of mTNFa IV (WT: n=8; KI n=7) compared to each other. The repeated measurements data were analyzed using a linear mixed model restricted maximum likelihood (REML). Covariance over time has been modeled as AR1 (autoregressive order 1) and variance heterogeneity across time points was accounted for. The significance of genotype effects on the changes in MAP and HR caused by the treatment were assessed by an F test.

Supplementary Table 9.

TELEMETRY	MAP				
**Fixed term**	**Wald statistic**	**n.d.f.**	**F statistic**	**d.d.f.**	**F pr**
**Time**	68.27	6	11.37	76.8	<0.001
**Genotype**	1.32	1	1.32	13.1	0.272
**Time.Genotype**	19.76	6	3.29	76.8	0.006

Statistical analysis of MAP obtained via telemetry, 5-35 min after injection of mTNFa IV (WT: n=8; KI n=7) compared to each other. The repeated measurements data were analyzed using a linear mixed model restricted maximum likelihood (REML). Covariance over time has been modeled as AR1 (autoregressive order 1) and variance heterogeneity across time points was accounted for. The significance of genotype effects on the changes in MAP and HR caused by the treatment were assessed by an F test.
